# Identification and validation of TSPAN13 as a novel temozolomide resistance-related gene prognostic biomarker in glioblastoma

**DOI:** 10.1371/journal.pone.0316552

**Published:** 2025-02-04

**Authors:** Haofei Wang, Zhen Liu, Zesheng Peng, Peng Lv, Peng Fu, Xiaobing Jiang

**Affiliations:** Department of Neurosurgery, Union Hospital, Tongji Medical College, Huazhong University of Science and Technology, Wuhan, China; Texas A&M University, UNITED STATES OF AMERICA

## Abstract

Glioblastoma (GBM) is the most lethal primary tumor of the central nervous system, with its resistance to treatment posing significant challenges. This study aims to develop a comprehensive prognostic model to identify biomarkers associated with temozolomide (TMZ) resistance. We employed a multifaceted approach, combining differential expression and univariate Cox regression analyses to screen for TMZ resistance-related differentially expressed genes (TMZR-RDEGs) in GBM. Using LASSO Cox analysis, we selected 12 TMZR-RDEGs to construct a risk score model, which was evaluated for performance through survival analysis, time-dependent ROC, and stratified analyses. Functional enrichment and mutation analyses were conducted to explore the underlying mechanisms of the risk score and its relationship with immune cell infiltration levels in GBM. The prognostic risk score model, based on the 12 TMZR-RDEGs, demonstrated high efficacy in predicting GBM patient outcomes and emerged as an independent predictive factor. Additionally, we focused on the molecule TSPAN13, whose role in GBM is not well understood. We assessed cell proliferation, migration, and invasion capabilities through in vitro assays (including CCK-8, Edu, wound healing, and transwell assays) and quantitatively analyzed TSPAN13 expression levels in clinical glioma samples using tissue microarray immunohistochemistry. The impact of TSPAN13 on TMZ resistance in GBM cells was validated through in vitro experiments and a mouse orthotopic xenograft model. Notably, TSPAN13 was upregulated in GBM and correlated with poorer patient prognosis. Knockdown of TSPAN13 inhibited GBM cell proliferation, migration, and invasion, and enhanced sensitivity to TMZ treatment. This study provides a valuable prognostic tool for GBM and identifies TSPAN13 as a critical target for therapeutic intervention.

## Introduction

Glioblastoma (GBM), the most prevalent and lethal malignant tumour of the central nervous system, is characterized by its highly invasive nature and a lack of effective therapeutic options, which result in its dismal prognosis. Patients with GBM typically have a median overall survival (OS) time of less than two years [1]. Currently, the standard treatment protocol involves maximal tumour resection followed by radiochemotherapy [2]. However, recent genome-wide molecular profiling studies have advanced our understanding of GBM tumorigenesis and chemoresistance, leading to the development of individualized therapies and novel therapeutic strategies [3]. These advancements in molecular pathology, including the identification of biomarkers such as IDH mutations [4], 1p19q codeletion [5], and MGMT promoter (MGMTp) methylation [6], have brought new hope to the field. The mutation status of IDH1/2 represents a critical feature in the molecular classification of gliomas. Patients with IDH mutations generally exhibit slower tumor progression and better prognosis. Additionally, drugs targeting IDH as a potential therapeutic target are currently under development [7,8]. The co-deletion of 1p/19q is an important diagnostic marker for grade II and III oligodendrogliomas and serves as a key criterion for distinguishing oligodendrogliomas from astrocytomas in the current WHO classification of gliomas. The 1p/19q co-deletion is associated with longer survival and is considered a favorable prognostic indicator in patients with oligodendroglioma. Gliomas with 1p/19q co-deletion show good responsiveness to radiotherapy and chemotherapy, particularly the PCV regimen [8,9]. MGMT promoter methylation is also one of the most commonly used prognostic markers in GBM. By suppressing MGMT expression, MGMT promoter methylation hinders DNA repair. This results in increased sensitivity of tumor cells to alkylating agents, such as TMZ. Patients with positive MGMT methylation exhibit a better response to TMZ treatment and have significantly prolonged survival [10]. The Cancer Genome Atlas (TCGA) project has classified glioblastoma into four major molecular subtypes based on gene expression profiles: Classical, Mesenchymal, Proneural, and Neural. Each subtype exhibits distinct clinical and molecular characteristics. The Proneural subtype, for instance, is associated with a better prognosis and is frequently characterized by G-CIMP positivity and mutations in isocitrate dehydrogenase (IDH) genes. In contrast, the Classical subtype is defined by epidermal growth factor receptor (EGFR) amplification and typically lacks IDH1/IDH2 mutations or 1p/19q co-deletion. The Mesenchymal subtype is marked by NF1 mutations or deletions, along with significant inflammation and immune cell infiltration, leading to the poorest prognosis among the subtypes [11]. Given the significant heterogeneity of GBM, further exploration of its molecular pathological subtypes and the identification of novel therapeutic targets are of great importance.

Temozolomide (TMZ), an oral anti-tumor drug approved by the FDA, is commonly used for glioblastoma treatment. As a DNA-alkylating agent, TMZ induces cell cycle arrest and apoptosis [12]. Temozolomide can cross the blood-brain barrier and has significantly improved overall survival in glioblastoma patients. However, approximately 50% of gliomas do not respond to TMZ due to intrinsic or acquired resistance, which is linked to factors such as p53 mutation, HFE mutation, and MGMT promoter demethylation [13]. The development of resistance to temozolomide limits the effectiveness of further treatment, resulting in higher recurrence rates and markedly poorer prognosis in patients with temozolomide resistance [13]. In recent years, several molecular targets, such as MGMT, PARP, and APE-1, have been identified as potential strategies to overcome temozolomide resistance in glioblastoma. Additionally, the combination of TMZ with other anticancer therapies, such as tamoxifen, rapamycin, and afatinib, has shown promise as an approach to combat TMZ resistance, demonstrating potential for clinical application [14]. This year, the combination of temozolomide and perifosine has also been discovered to synergistically inhibit glioblastoma by blocking DNA repair and inducing apoptosis [15]. Therefore, a comprehensive analysis of TMZ resistance-related genes can enhance the understanding of the mechanisms underlying TMZ resistance in glioblastoma. This approach is valuable for identifying novel prognostic biomarkers and developing effective therapeutic strategies for GBM.

In this study, we identified and validated prognostic TMZ resistance-related differentially expressed genes (TMZR-RDEGs) and constructed a novel prognostic model for GBM based on 12 TMZR-RDEGs. This model demonstrated excellent predictive performance for GBM patient prognosis, and the risk score calculated based on this model was identified as an independent risk factor. This risk score was significantly associated with various biological responses and pathways, as well as with the gene mutation burden and immune cell infiltration. TSPAN13 (Tetraspanin 13), identified as one of the 12 TMZ resistance-related differentially expressed genes (TMZR-RDEGs) in our study, has been reported to play a role in the progression of various cancers [16–18]. Among these 12 TMZR-RDEGs, TSPAN13 is the only gene that has not yet been specifically investigated in glioma, making it the focal target for our subsequent research. Focusing on Tetraspanin-13 (TSPAN13), we found that TSPAN13 was upregulated in GBM tissues and that its knockdown significantly inhibited GBM cell proliferation, migration, and invasion. Furthermore, TSPAN13 knockdown enhanced TMZ sensitivity in vitro and in vivo, and increasing DNA damage level, highlighting the potential of TSPAN13 as a therapeutic target in GBM treatment.

## Materials and methods

### Data acquisition

Datasets related to temozolomide (TMZ) resistance, namely, GSE199689, GSE193957 [19], and GSE100736, were acquired from the Gene Expression Omnibus (GEO, https://www.ncbi.nlm.nih.gov/gds). The GSE199689 and GSE193957 datasets [19] each comprise data for three samples from a TMZ-resistant U87 cell line and three samples from a TMZ-sensitive U87 cell line. Similarly, the GSE100736 dataset comprises data for three samples of a TMZ-resistant U251 cell line and three samples of a TMZ-sensitive U251 cell line. As indicated by the data processing information provided by the data submitters, the three datasets have undergone quantile normalization. Furthermore, data quality control was conducted on each dataset using the ‘arrayQualityMetrics’ R package to ensure robust and reliable data analysis [20]. When multiple probes corresponded to the same gene symbol, the mean value of these probes was used. Each dataset was initially analyzed independently to identify differentially expressed genes. Batch effect correction for the three distinct gene expression microarray datasets was performed using the ComBat function from the ‘sva’ R package [21]. Following integration, the Robust Rank Aggregation (RRA) method was employed to prioritize DEGs [22], enabling the identification of cross-platform consistent genes through rank aggregation across different platforms.

RNA sequencing (RNA-seq) data and clinical information for glioma patients were obtained from the Chinese Glioma Genome Atlas (public) [23] (CGGA; http://www.cgga.org.cn/) and The Cancer Genome Atlas (TCGA; https://portal.gdc.cancer.gov/). Patients who lacked survival data, had an overall survival time of less than 30 days, or did not have a definitive histopathological diagnosis were excluded. Additionally, 168 patients represented in the TCGA-GBM dataset were chosen to establish the training cohort. We utilized CGGA mRNAseq_325 [24] and CGGA mRNAseq_693 [25] from the CGGA database as external independent datasets for validation, aiming to assess the applicability and generalizability of our prognostic model across different populations. For the validation cohort, 128 patients diagnosed with GBM diagnosis represented in the CGGA mRNAseq_325 dataset (CGGA325-GBM) and 237 patients diagnosed with GBM represented in the CGGA mRNAseq_693 dataset (CGGA693-GBM) were selected. The obtained gene expression matrix data have been pre-mapped to gene IDs. All RNA-seq data were standardized as fragments per kilobase of exon model per million mapped reads (FPKM) values.

### Identification of differentially expressed genes (DEGs) from the GEO datasets

Utilizing gene expression data from the GSE199686, GSE193957, and GSE100736 datasets, we applied the ‘limma’ package in R software for differential expression analysis to identify differentially expressed genes (DEGs). The selection criteria were set as a log_2_ | fold change (FC) | of > 1 and an adjusted P value of <0.001. To visualize these DEGs, the ‘ggplot2’ and ‘ggrepel’ packages in R were used to generate volcano plots.

### Robust rank aggregation analysis

To minimize inconsistencies and to integrate the results from several microarray studies, the robust rank aggregation (RRA) method, which is an effective tool for integrating the results of multiple array analyses, was adopted to identify robust DEGs [22,26]. Before RRA analysis, we obtained lists of up- and downregulated genes for each dataset, which were generated by calculating the expression fold changes between the TMZ-resistant cells and the control cells. The “Robust Rank Aggregation” R package was used to integrate all the ranked gene lists generated from the datasets. The adjusted P value in the RRA package indicates the possibility of a high rank for each gene in the final gene list. After RRA analysis, 194 TMZ resistance-related differentially expressed genes (TMZR-RDEGs) were screened for subsequent prognostic model construction.

### Construction and validation of the TMZR-RGPI

Univariate Cox regression analysis was also conducted to identify prognostic TMZR-RDEGs in the TCGA-GBM cohort. This analysis identified 48 differentially expressed genes (P value <  0.05), which were identified as prognostic TMZR-RDEGs. In the training cohort, the prognostic TMZR-RDEGs were integrated into a Cox regression model with the least absolute shrinkage and selection operator (LASSO) penalty utilizing the ‘glmnet’ R package [27]. To determine the optimal lambda (λ) value, a minimum tenfold cross-validation approach was employed. This process led to the identification of 12 TMZR-RDEGs (including SLC43A3, IGFBP6, BDKRB1, TSPAN13, MMP2, MAPRE3, HTRA1, MDK, MME, PTX3, PTPRN2, and FLNC), which were subsequently used to develop the TMZ Resistance-Related Gene Prognostic Index (TMZR-RGPI). The construction of this prognostic index was based on the formula derived from the Cox proportional hazards regression model.


$$h\left(t\right)=\;h_{0}\;\left(t\right)\text{*exp }\left({\text{β}}_{1}{\text{X}}_{1}+{\text{ β}}_{2}{\text{X}}_{2}\;+\cdots +{\text{ β}}_{n}{\text{X}}_{n}\right)\;$$

Subsequently, we applied a logarithmic transformation to the results of this formula to construct our TMZR-RGPI model.


$$\text{TMZR}-\text{RGPI}=lnh_{0}\;\left(t\right)+\left({\text{β}}_{1}{\text{X}}_{1}+{\text{ β}}_{2}{\text{X}}_{2}\;+\cdots +{\text{ β}}_{n}{\text{X}}_{n}\right)$$

In the formula for calculating the TMZR-RGPI, X_1_, X_2_, …, X_n_ refers to the expression levels of the selected TMZR-RDEGs, while β_1_, β_2_, …, β_n_ refers to the corresponding coefficients in the Cox proportional hazards regression model. The term h_0_(t) denotes the baseline hazard function, which is assumed to be a constant in this model. We used the median TMZR-RGPI as a cut-off to stratify patients into the high- and low-TMZR-RGPI subgroups. Kaplan-Meier survival curves, along with the log-rank test results, were plotted using the ‘survminer’ R package to compare overall survival (OS) between these subgroups. Furthermore, receiver operating characteristic (ROC) curve analysis was employed to evaluate the prognostic accuracy of the TMZR-RGPI utilizing the ‘timeROC’ R package [28]. The CGGA693-GBM and CGGA325-GBM cohorts were used to validate our prognostic model.

### Establishment and evaluation of a nomogram

Univariate and multivariate Cox regression analyses were conducted to ascertain the independent prognostic significance of the TMZR-RGPI. A nomogram incorporating the independent prognostic factors identified in the training cohort was subsequently developed using the ‘rms’ R package. To graphically evaluate the nomogram’s performance, calibration curves for 1-, 2-, and 3-year survival predictions were plotted. The predictive accuracy of the nomogram was assessed using the concordance index (C-index) and receiver operating characteristic (ROC) curve analysis [29].

### Gene Ontology (GO) and pathway enrichment analyses based on our prognostic model

Initially, the ‘limma’ R package was used to identify DEGs between the high- and low-TMZR-RGPI subgroups within the TCGA-GBM cohort. The criteria for DEG identification were set as an absolute log_2_-fold change (|log_2_FC|) of > 1 and an adjusted p value of < 0.05. Gene Ontology (GO) analysis, encompassing the biological process (BP), cellular component (CC), and molecular function (MF) categories, was subsequently conducted using the ‘ClusterProfiler’ R package based on the identified DEGs. Furthermore, pathway enrichment analysis based on the KEGG and HALLMARK gene sets was performed through the gene set enrichment analysis (GSEA) method [30], also with the ‘ClusterProfiler’ R package. Threshold criteria of an adjusted p value of < 0.05, a q value of < 0.05, and an absolute normalized enrichment score (NES) of >  1 were adopted to determine significance.

### Immune infiltration analysis

We utilized the ESTIMATE algorithm via the ‘estimate’ package in R to calculate the ImmuneScore, StromalScore, and ESTIMATEScore for each sample. Additionally, the infiltration levels of 24 types of immune cells in each sample were assessed using the single-sample GSEA approach implemented via the ‘GSVA’ package in R [31].

### The mutation profile and TMB analysis

We retrieved somatic mutation data from the TCGA-GBM cohort utilizing the GDCquery_Maf() function of the ‘TCGAbiolinks’ package in R. Analysis and visualization of the top 20 individual cell variants with the highest mutation frequency across the high- and low-TMZR-RGPI groups were conducted using the ‘maftools’ package. The tumour mutational burden (TMB) is defined as the total number of nonsynonymous mutations per megabase of the genomic sequence. These mutations include somatic mutations, coding mutations, silent mutations, base substitutions, and insertions or deletions. The somatic variant data were formatted using the mutation annotation format (MAF). Subsequently, we employed the ‘maftools’ R package to explore the differences in somatic variants between the two TMZR-RGPI subgroups [32].

### Cell culture and transfection

The human glioma cell lines U251 and U87 were purchased from the Cell Bank Type Culture Collection of the Chinese Academy of Sciences (Shanghai, China) and identified by Procell Life Science & Technology Co., Ltd (Wuhan, China). The cells were cultured in Dulbecco’s modified Eagle’s medium (DMEM; Cytiva, USA) supplemented with 10% fetal bovine serum (Gibco, Thermo Fisher Scientific, USA), penicillin (100 U/mL) and streptomycin (100 μg/mL) at 37°C in a humidified atmosphere of 5% CO_2_. The cell source and culture conditions are consistent with our previous published articles [33]. TMZ was obtained from MedChemExpress Corporation (New Jersey, USA). All subsequent cell experiments and qRT-PCR data were generated in our laboratory as part of this study to validate the model’s predictive results.

All small interfering RNAs (siRNAs) against the target genes and negative control siRNAs were synthesized by Genomeditech (Shanghai, China). U87 and U251 cells were transfected using Lipofectamine 2000 Transfection Reagent (Invitrogen, Thermo Fisher Scientific, USA) according to the manufacturer's instructions. In brief, U87 and U251 cells were seeded into 24-well plates and allowed to reach 50–70% confluence. TSPAN13 siRNA (0.5 µg/well) and Lipo 2000 (2 µl/well) were diluted separately in Opti-MEM (50 µl/well). The diluted siRNA and liposomes were then mixed and incubated at room temperature for 10 minutes before being added to the cultured U87 and U251 cells. After 24 hours, the medium was replaced with regular complete medium, and the cells were further cultured for 3 days for subsequent experimental validation. The sequences of the TSPAN13 siRNAs used were as follows: TSPAN13-si-1 F 5’-AAUUAGCAGCAGACUAACCAA, 3’-GGUUAAUCGUCGUCUGAUUGG; TSPAN13-si-2 F 5’-UAAUCAUAUAAAAAAAUAGCA, 3’-UUAUUAGUAUAUUUUUUUAUC.

### Quantitative real-time polymerase chain reaction (qRT–PCR)

Total RNA was isolated from each transfected U87 and U251 cell lines employing TRIzol agent (Invitrogen). Following the provided guidelines, cDNA synthesis was achieved through reverse transcription, utilizing a kit from Takara (RR036A). The analysis via qRT-PCR was subsequently executed on a LightCycler 480 System, employing TB Green® Premix Ex Taq™ II (Takara RR820A) for amplification. For data normalization, GAPDH served as the reference, and mRNA levels were quantified using the comparative Ct approach (^ΔΔ^Ct). Primer sequences were procured from Accurate Biotechnology (Wuhan, China). The sequences of the primers used were as follows: GAPDH, forward, 5′-GGAAGCTTGTCATCAATGGAAATC-3′ and reverse, 5′-TGATGACCCTTTTGGCTCCC-3′; TSPAN13, forward, 5′- GGTTAGTCTGCTGCTAATTG -3’ and reverse, 5′- TCAGACCCACTAAAGCAATC -3’.

### Immunofluorescence and immunohistochemical analyses of glioma tissue microarrays

The glioma tissue microarray, along with the corresponding clinical data, was provided by Professor Junhui Liu from Renmin Hospital of Wuhan University. The development and application of this tissue microarray were approved by the Ethics Committee of Wuhan University, and all research were conducted under appropriate guidelines and written informed consent was obtained from all glioma tissue providers. Research related to glioma tissue adhered strictly to the principles outlined in the Declaration of Helsinki. Ethics approval was obtained from the ethics committee at Wuhan Union Hospital [2019] IEC (8561). Informed consent was obtained from all subjects and/or their legal guardians. For immunohistochemical (IHC) analysis, formalin-fixed paraffin-embedded tissue sections and tissue microarrays were used. The process involved deparaffinizing and hydrating the sections, followed by antigen retrieval in 10 mM sodium citrate solution. Endogenous peroxidase activity was quenched using 3% hydrogen peroxide (H_2_O_2_) for 10 minutes. The sections were then incubated overnight with primary antibodies (anti- TSPAN13, 18974-1-AP, ProteinTech, China) and then with horseradish peroxidase (HRP)-conjugated secondary antibodies. Signals were detected using diaminobenzidine (DAB) staining, and the sections were counterstained with haematoxylin for visualization. Similar processing was also described in our previous article [33].

Cells underwent cultivation on coverslips coated with collagen and received temozolomide treatment as specified. Following treatment, the cells were fixed with 4% paraformaldehyde and then incubated with 50 μM NH_4_Cl. Blocking was performed using 3% bovine serum albumin (BSA) prior to overnight incubation at 4°C with primary anti-γ-H2A.X antibody (HL1299, GeneTex, Shenzhen, China). After washing with PBS, the cells were incubated with a fluorescent secondary antibody (CoraLite594-conjugated goat anti-rabbit IgG, SA00013-4, ProteinTech, Wuhan, China). Subsequently, the prepared coverslips were affixed to slides with the aid of ProLong Gold antifade agent (P36391, Invitrogen), which is enriched with 4′,6-diamidino-2-phenylindole (DAPI) for nuclear staining. Images for Immunofluorescence (IF) and Immunohistochemistry (IHC) were captured using an Olympus IX73 inverted microscope. The analysis of protein levels was conducted using ImageJ software, where the expression is denoted as ratio values, specifically average optical density (AOD) Figs, calculated by dividing the integrated optical density (IOD) by the positively stained area.

### Western blotting

Cell lysates from U251 and U87 lines were obtained through brief sonication within a modified RIPA solution (Biosharp, China), enhanced with inhibitors for proteinases and phosphatases. Protein levels within these lysates were quantified employing a BCA protein assay kit (Beyotime Biotechnology, China), adhering to the protocol provided by the manufacturer. Following this, protein separation was achieved through 10% SDS-PAGE, with the proteins then being transferred to a PVDF membrane (Millipore, USA). The membrane was blocked with 5% non-fat milk at room temperature for 2 hours and was then incubated overnight at 4°C with primary antibodies (anti-γ-H2A.X HL1299, GeneTex; anti-GAPDH 60004-1-Ig, anti-Erk1/2 11257-1-AP, anti-pErk1/2 28733-1-AP and anti- TSPAN13, 18974-1-AP, ProteinTech, China; anti-JAK2 #3230, anti-pJAK2 #3774, anti-STAT3 #12640, anti-pSTAT3 #9145, CST). After three washes with 1 × TBST, the membrane was incubated with appropriate horseradish peroxidase-conjugated secondary antibodies (SA0001-1 or SA0001-2, ProteinTech, China) for 2 hours. The immunoreactions were visualized using ECL reagent, and the immunoblots were developed to detect the target proteins.

### Cell proliferation and TMZ sensitivity assays

The proliferation of cells and their responsiveness to TMZ were assessed using a Cell Counting Kit-8 (CCK-8; Beyotime, C0038). Following a 48-hour transfection period, cells were plated at a density of 5,000 cells per well in 96-well plates and incubated for periods of 24, 48, or 72 hours. Subsequently, each well received 10 ml of CCK-8 solution, and cell proliferation was quantified by measuring the absorbance at 450 nm. In assessing TMZ sensitivity, cells were initially seeded at 10,000 cells per well and allowed to adhere for 12 hours. Varying concentrations of TMZ were then introduced, followed by the addition of CCK-8 solution 48 hours later to evaluate cell viability.

A 5-ethynyl-2’-deoxyuridine (EdU) incorporation assay was used to assess the proliferation of transfected U251 and U87 cells. Experimental procedures were performed according to the instructions of the BeyoClick™ EdU-488 kit (Beyotime Biotechnology, China). After cells had adhered to a 96-well plate, they were incubated with 10 μM EdU for 4 hours. Subsequently, the cells were fixed, permeabilized, and stained with an Alexa Fluor 488 reaction cocktail for EdU detection and with Hoechst for nuclear labelling. The stained samples were then visualized and imaged using a fluorescence microscope Olympus IX73 to observe. The image statistics were analyzed and calculated by ImageJ software.

### Invasion and migration assays

To evaluate the invasive capabilities of the cells, Transwell assays were performed. Initially, cells were deprived of serum for 24 hours to eliminate any effects of proliferation on the invasion outcomes. In the invasion assays, a defined quantity of glioma cells was placed on top of Matrigel-precoated membranes within the upper chambers of Transwell setups (Corning, USA). The bottom chambers were supplemented with 500 μl of DMEM containing 10% FBS to act as a chemoattractant. After incubating for 24 hours at 37°C, non-invading cells remaining on the upper side of the membranes were fixed using 4% paraformaldehyde for 15 minutes and then stained with 0.1% crystal violet for another 15 minutes.

In the wound healing experiment, cells were plated in 6-well plates and allowed to reach roughly 90% confluence. A sterile pipette tip then introduced a straight-line gap (the ‘wound’) across the cell layer. Post-wounding, cells were maintained in serum-free DMEM to continue growth for 24 hours. The healing progress was documented with an inverted microscope, and the extent of wound closure was quantitatively assessed through ImageJ software, measuring the area of gap reduction.

### Tumor-bearing mouse experiments

The animal study was approved by the Institutional Animal Care and Use Committee of Huazhong University of Science and Technology. Four-week-old male BALB/c-nu mice were obtained from Hubei Bainte Biotechnology Co. Ltd and housed in the animal facilities of Huazhong University of Science and Technology under appropriate conditions. All mice were anesthetized with an intraperitoneal injection of a Ketamine and xylazine cocktail before surgery. To establish the experimental model, 1 × 10^5^ U251 cells were transplanted into the right frontal lobe of the four-week-old mice. The body weight of the mice was recorded every five days, and their survival time was monitored until day 50. After the mice died, their brain tissues were collected for HE staining.

In this study, all mice were housed in SPF-grade sterile isolation units to ensure a pathogen-free environment and maintain the health status of the animals. The animal study was approved by the Institutional Animal Care and Use Committee of Huazhong University of Science and Technology [2022] IACUC (3854). All experiments were performed in accordance with relevant guidelines and regulations. Throughout the experiment, researchers conducted daily health assessments of the mice, with a focus on monitoring activity levels and behavioral changes. The criteria for euthanasia were defined as a significant reduction in activity or prolonged immobility, accompanied by severe weakness and lethargy. Mice meeting these criteria were humanely euthanized using carbon dioxide (CO₂) asphyxiation, in accordance with ethical standards. Four mice were found dead prior to scheduled euthanasia. The total observation period was 50 days, at the end of which all remaining mice were euthanized. The causes of death for those that died prior to this time point were thoroughly documented and analyzed. All personnel involved in the experiment received standard training in laboratory animal handling to ensure compliance with experimental protocols and the welfare of the animals.

## Results

### Prognostic TMZR-RDEG identification by RRA-based integrated analysis

**Fig 1:**
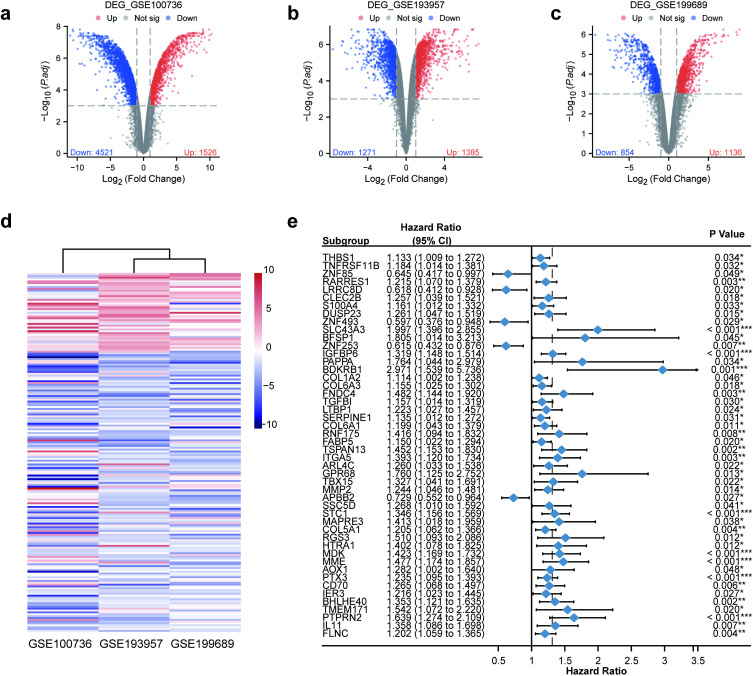
Identification of prognostic TMZR-RDEGs in GBM. (a-c) The volcano plot shows the differentially expressed genes in GSE199689, GSE193957, and GSE100736 datasets. (d) The heatmap visualizes the expression levels of 196 TMZR-RDEGs across various datasets, identified via RRA methods. Red and blue bands represent high and low gene expressions, respectively. (e) The forest plot showed the 48 prognostic TMZR-RDEGs in the TCGA-GBM dataset. (*p <  0.05, **p <  0.01, ***p <  0.001).

Three datasets related to temozolomide-resistant glioblastoma (GSE199689, GSE193957, and GSE100736) obtained from the GEO database were included in this study. Differential expression analysis was performed separately for each dataset. A total of 6047 DEGs were obtained from the GSE100736 dataset, 2656 DEGs were obtained from the GSE193957 dataset, and 1990 DEGs were obtained from the GSE199689 dataset; all the DEGs were visualized in a volcano plot (Fig 1a–1c). In the RRA method, genes with lower p values have higher ranks and credibility of differential expression, and the significance score provides a rigorous approach to retain the statistically relevant genes. The genes overlapping among all three gene sets and having a significance score of < 0.05 were identified as TMZ resistance-related differentially expressed genes (TMZR-RDEGs). Through the RRA method, 43 upregulated TMZR-RDEGs and 153 downregulated TMZR-RDEGs were identified, and the full results are shown in S1 Table and the heatmap in Fig 1d. Furthermore, to investigate the prognostic value of TMZR-RDEGs in GBM patients, univariate Cox regression analysis was performed based on the TCGA-GBM dataset. Among the TMZR-RDEGs (3 genes did not have a match in the TCGA-GBM cohort), 48 DE-MRGs – 5 genes with a hazard ratio (HR) of <1 and 43 genes with a HR of >1–were significantly associated with overall survival (OS) in patients with GBM in the TCGA-GBM dataset (Fig 1e, S2 Table). These results indicate that our TMZR-RGPI model can reliably predict the prognosis of patients with glioblastoma, especially in terms of survival beyond 1 year.

### Construction and validation of the TMZR-RGPI

**Fig 2:**
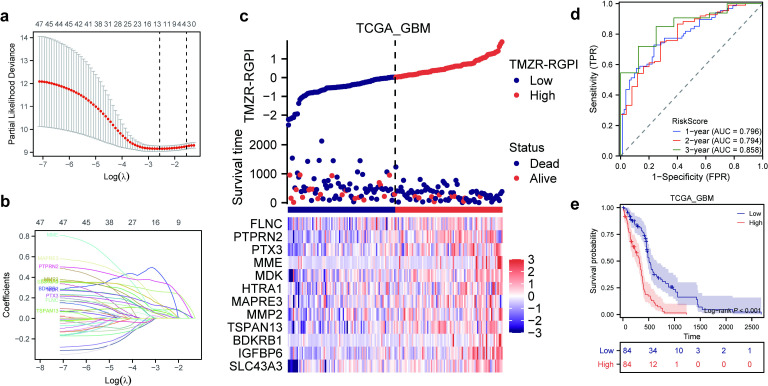
Construction of the TMZR-RGPI in the TCGA-GBM cohort. (a, b) LASSO regression was performed with the minimum criteria. (c) The distribution plots of TMZR-RGPI, survival status and expression of 12 selected TMZR-RDEGs. (d) Time-Dependent ROC Curves of the 12-Gene TMZR-RGPI Model in the TCGA GBM Cohort. (e) Kaplan‒Meier curves of TMZR-RGPI subgroups for survival.

In our analysis, the 48 prognostic TMZR-RDEGs identified in the TCGA-GBM cohort were integrated into a regression model with the least absolute shrinkage and selection operator (LASSO) penalty (as depicted in Fig 2a, 2b). This led to the selection of 12 prognostic TMZR-RDEGs (SLC43A3, IGFBP6, BDKRB1, TSPAN13, MMP2, MAPRE3, HTRA1, MDK, MME, PTX3, PTPRN2, and FLNC) for construction of the TMZR-RGPI model. The TMZR-RGPI score was calculated using the following formula: TMZR-RGPI =  −9.239 (value of ln[h_0_(t)]) +  (0.325 *  expression level of SLC43A3) +  (0.075 *  expression level of IGFBP6) +  (0.697 *  expression level of BDKRB1) +  (0.140 *  expression level of TSPAN13) +  (0.133 *  expression level of MMP2) +  (0.420 *  expression level of MAPRE3) +  (0.225 *  expression level of HTRA1) +  (0.163 *  expression level of MDK) +  (0.108 *  expression level of MME) +  (0.115 *  expression level of PTX3) +  (0.400 *  expression level of PTPRN2) +  (0.088 *  expression level of FLNC). Patients were then stratified into subgroups based on the median value of the TMZR-RGPI as the cut-off. Within the TCGA-GBM cohort, analysis of the TMZR-RGPI and survival status distributions revealed that patients with higher TMZR-RGPI scores tended to have shorter overall survival (OS) times and a higher incidence of death (Fig 2c). The receiver operating characteristic (ROC) curves demonstrated the satisfactory predictive performance of the TMZR-RGPI, with area under the curve (AUC) values for 1-year, 2-year, and 5-year survival of 0.796, 0.794, and 0.858, respectively (Fig 2d). Additionally, Kaplan‒Meier survival analysis indicated that patients in the low-TMZR-RGPI subgroup had significantly longer overall survival (OS) times than did those in the high-TMZR-RGPI subgroup (Fig 2e). To further validate the prognostic efficacy of the TMZR-RGPI, analogous analyses were conducted in the CGGA693-GBM and CGGA325-GBM cohorts. Consistent with the results observed in the training cohort, patients with a lower TMZR-RGPI consistently exhibited better survival outcomes than did those with a higher TMZR-RGPI in these validation cohorts (S1a, b, d, and e Fig). The receiver operating characteristic (ROC) curves for the CGGA693-GBM cohort reinforced the robust predictive ability of the TMZR-RGPI for overall survival (OS), with area under the curve (AUC) values for 1-year, 2-year, and 3-year survival of 0.610, 0.791, and 0.789, respectively (S1c Fig). Similarly, in the CGGA325-GBM cohort, the TMZR-RGPI demonstrated good predictive accuracy, with a 1-year AUC of 0.687, a 2-year AUC of 0.784, and 3-year AUC of 0.724 (S1f Fig).

### Stratification analysis of the prognostic TMZR-RGPI model based on clinical characteristics

**Fig 3:**
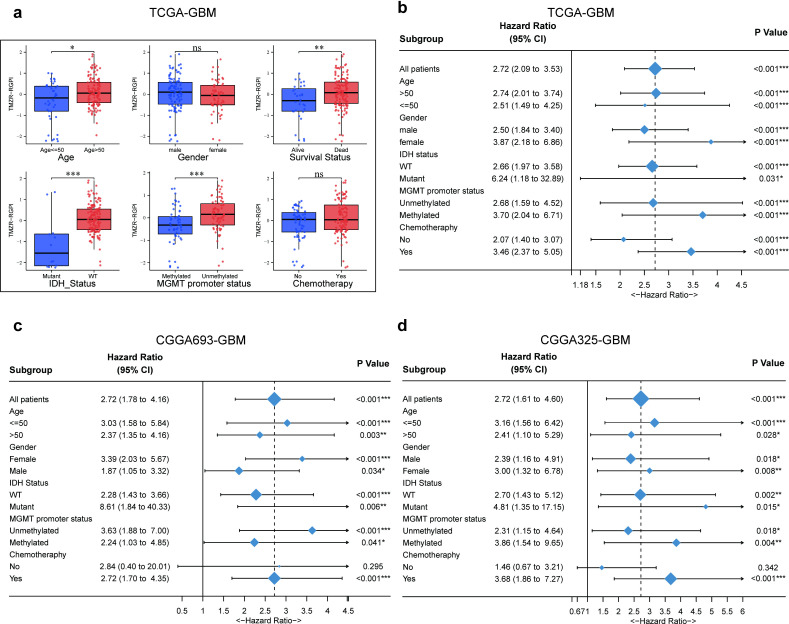
Correlation analysis of TMZR-RGPI with clinicopathological features in training and validation cohorts. (a) Variations in TMZR-RGPI among glioma patients categorized by age, sex, grade, survival status, IDH status, MGMT promoter status, and whether to receive chemotherapy. (b-d) Forest plots illustrating survival outcomes in subgroups stratified by these clinicopathological characteristics. (*p <  0.05, **p <  0.01, ***p <  0.001, ns =  not significant).

In our study, we compared the TMZR-RGPI score among patients stratified by various clinical characteristics, including age, sex, tumour grade, survival status, IDH status, MGMT promoter methylation status, and chemotherapy status. Within the TCGA-GBM cohort, patients characterized by clinical features such as an age of greater than 50, death, wild-type IDH status, and unmethylated MGMT promoter methylation status had significantly higher TMZR-RGPI scores. Conversely, no significant differences in the TMZR-RGPI score were observed between patients stratified by sex or chemotherapy status (Fig 3a). To evaluate whether various clinical characteristics influence the prognostic accuracy of the TMZ resistance-related gene prognostic index (TMZR-RGPI), we conducted subgroup survival analyses. The results of these analyses were visualized using forest plots. In the training cohort, patients with a high TMZR-RGPI consistently exhibited poorer survival outcomes than did those with a low TMZR-RGPI across all subgroups (Fig 3b). In the validation cohorts, namely, CGGA693-GBM and CGGA325-GBM, the results of these subgroup analyses largely mirrored those in the training cohort. However, an exception was noted in the subgroup of patients who did not receive chemotherapy (Fig 3c, 3d). In alignment with these findings, Kaplan‒Meier (KM) survival analysis was performed in the training cohort and the validation cohorts to assess the impact of the TMZR-RGPI score on GBM patients across these clinical characteristics (S2a-d Fig). Subsequently, for further exploration, KM survival analysis based on the TMZR-RGPI score was performed in the subgroup of patients who received radiotherapy (S3e, f Fig). This analytical approach allowed a comprehensive evaluation of the prognostic significance of the TMZR-RGPI score in different clinical contexts within the GBM patient population.

### Construction and evaluation of a nomogram

**Fig 4:**
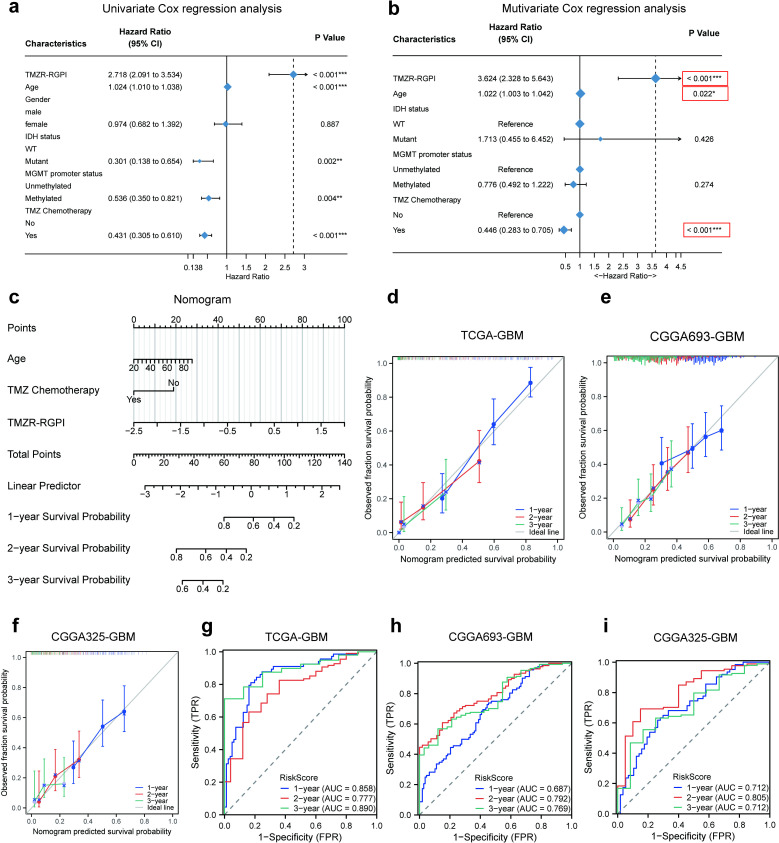
Establishment and evaluation of a nomogram. (a, b) Conducted univariate and multivariate Cox regression analyses within the TCGA-GBM cohort. (c) Nomogram based on TMZR-RGPI, age and whether to receive chemotherapy. (d-f) Calibration curves showed the concordance between predicted and observed 1-, 2-, and 3-year OS in TCGA-GBM, CGGA693-GBM, and CGGA325-GBM. (g-i) ROC curve analyses of the nomogram in predicting 1-, 2-, and 3-year OS in TCGA-GBM, CGGA693-GBM, and CGGA325-GBM. (*p <  0.05, **p <  0.01, ***p <  0.001, ns =  not significant).

To ascertain whether the established TMZR-RGPI could serve as a reliable prognostic predictor for glioma, we carried out univariate and multivariate Cox regression analyses incorporating common clinicopathological characteristics. In the training cohort, the TMZR-RGPI demonstrated satisfactory prognostic efficacy in combination with factors such as age, IDH status, MGMT promoter methylation status, and TMZ chemotherapy status (Fig 4a). Additionally, the TMZR-RGPI score was identified as an independent predictor in the multivariate Cox regression analysis (Fig 4b), underscoring its potential as a novel and robust prognostic biomarker. To improve the clinical applicability of our prognostic TMZR-RGPI model, we developed a nomogram integrating these independent prognostic factors (age, TMZ chemotherapy status, and TMZR-RGPI score) within the training cohort (Fig 4c). Internal validation of the nomogram involved the calculation of the concordance index (C-index) and the use of calibration plots, while external validation was performed in the validation cohorts using similar methods. The C-index of this nomogram was determined to be 0.754 in the TCGA-GBM cohort, 0.638 in the CGGA693-GBM cohort, and 0.669 in the CGGA325-GBM cohort. The calibration plots demonstrated excellent agreement between the actual outcomes and the probabilities predicted by the nomogram for 1-, 2-, and 3-year overall survival (OS) in both the training and validation cohorts (Fig 4d, 4e and 4f). The receiver operating characteristic (ROC) curves highlighted the impressive sensitivity and specificity of the prognostic TMZR-RGPI model in the TCGA-GBM cohort (1-year AUC =  0.858, 2-year AUC =  0.777, 3-year AUC =  0.890; Fig 4g), CGGA693-GBM cohort (1-year AUC =  0.687, 2-year AUC =  0.792, 3-year AUC =  0.769; Fig 4h), and CGGA325-GBM cohort (1-year AUC =  0.712, 2-year AUC =  0.805, 3-year AUC =  0.712; Fig 4i). Collectively, these results substantiated the satisfactory prognostic efficacy of the nomogram for glioblastoma, indicating its potential as a quantitative tool for predicting the prognosis of glioblastoma patients, particularly with respect to survival beyond 1 year.

### Mutation profiles of the TMZR-RGPI subgroups

To further investigate the mechanisms associated with the TMZR-RGPI model in glioblastoma, we analyzed the genetic mutation profile within the TCGA_GBM cohort. This led to the identification of the 20 genes with the highest mutation rates across the TMZR-RGPI subgroups. While there was no significant difference in the overall mutation frequency, the mutation frequencies of specific genes varied considerably between the low- and high-TMZR-RGPI subgroups. Notably, mutations in TTN, SPTA1, RYR2, and PKHD1 were more prevalent in the high-TMZR-RGPI subgroup. Conversely, TP53, RB1, FLG, and ATRX had higher mutation frequencies in the low-TMZR-RGPI subgroup (S3a Fig). Furthermore, a negative correlation was observed between the tumour mutational burden (TMB) and the TMZR-RGPI score, with the low-TMZR-RGPI subgroup exhibiting a significantly greater TMB than the high-TMZR-RGPI subgroup (S3b Fig).

### Differential expression and functional enrichment analyses of the TMZR-RGPI subgroups

Differential expression and functional enrichment analyses were also conducted to further explore the differences between the TMZR-RGPI subgroups. As illustrated in S4a Fig, a total of 205 differentially expressed genes (DEGs), namely, 191 upregulated and 14 downregulated genes, were identified between the high- and low-TMZR-RGPI subgroups. Gene Ontology (GO) enrichment analysis revealed that 95 biological process (BP) terms, 18 cellular component (CC) terms, and 13 molecular function (MF) terms were significantly enriched in these DEGs (adjusted p value <  0.001; S3 Table). The enriched BP terms included response to chemical, immune system process, extracellular matrix organization, regulation of signalling receptor activity, and defense response (S4b Fig). The significantly enriched CC terms were extracellular matrix, collagen trimer, vesicle lumen, secretory vesicle, and cytoplasmic vesicle lumen (S4c Fig). The enriched MF terms were receptor ligand activity, cytokine activity, molecular function regulator, G protein-coupled receptor binding, and growth factor activity (S4d Fig). Additionally, gene set enrichment analysis (GSEA) indicated enrichment not only of cancer-related pathways, such as epithelial-mesenchymal transition, hypoxia, cell cycle, P53 pathway, and KRAS signaling, but also of immune-related pathways, including inflammatory response, cytokine-cytokine receptor interaction, and adhesion molecules (cams) in the high-TMZR-RGPI subgroup (S4e,f Fig).

### Correlation between the TMZR-RGPI score and immune cell infiltration in GBM

**Fig 5:**
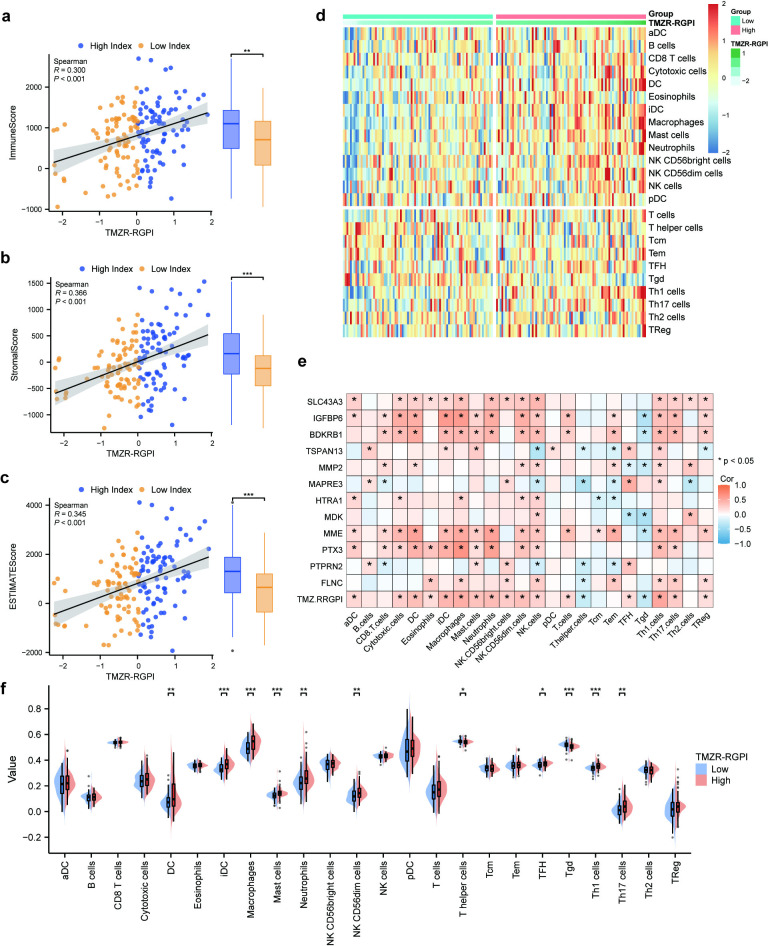
Association of TMZR-RGPI with Immune Cell Infiltration. (a) Scatter plot illustrating the positive correlation between TMZR-RGPI and ImmuneScore (Spearman’s rank correlation coefficient). (b) Scatter plot demonstrating the positive correlation between TMZR-RGPI and StromalScore (Spearman’s rank correlation coefficient). (c) Scatter plot depicting the positive correlation between TMZR-RGPI and ESTIMATEScore (Spearman’s rank correlation coefficient). (d) Heatmap displaying the association between TMZR-RGPI and the infiltration levels of 24 immune cell types in the TCGA-GBM dataset. (e) Correlation matrix of immune infiltration and TMZR-RGPI, along with the majority of its constituent molecules. (f) Boxplots revealing the relationship between TMZR-RGPI levels and infiltration levels of 24 immune cell types in the TCGA-GBM cohort. (*p <  0.05, **p <  0.01, ***p <  0.001).

Given that the functional enrichment analysis linked the TMZR-RGPI score to immune-related processes and pathways, we further investigated the association between TMZR-RGPI and the tumour immune microenvironment in GBM. Initially, we examined the correlation between the TMZR-RGPI and ESTIMATE scores, including the ImmuneScore, StromalScore, and ESTIMATEScore, calculated using the ESTIMATE algorithm within the TCGA-GBM cohort. The findings indicated significant positive correlations between the TMZR-RGPI score and the ESTIMATE scores in TCGA-GBM datasets (Fig 5a–c). Furthermore, we assessed the infiltration levels of 24 immune cell types in the TCGA-GBM cohort using the single-sample gene set enrichment analysis (ssGSEA) algorithm (Fig 5d). Our subsequent analysis revealed that TMZR-RGPI and the majority of its constituent molecules were correlated with immune infiltration in GBM tumor tissues (Fig 5e). Additionally, we observed that in the TCGA-GBM cohort, nearly half of the immune cell types exhibited significant differences in infiltration between the high- and low-TMZR-RGPI subgroups (Fig 5f).

### TSPAN13 is highly expressed in glioblastoma patients

**Fig 6:**
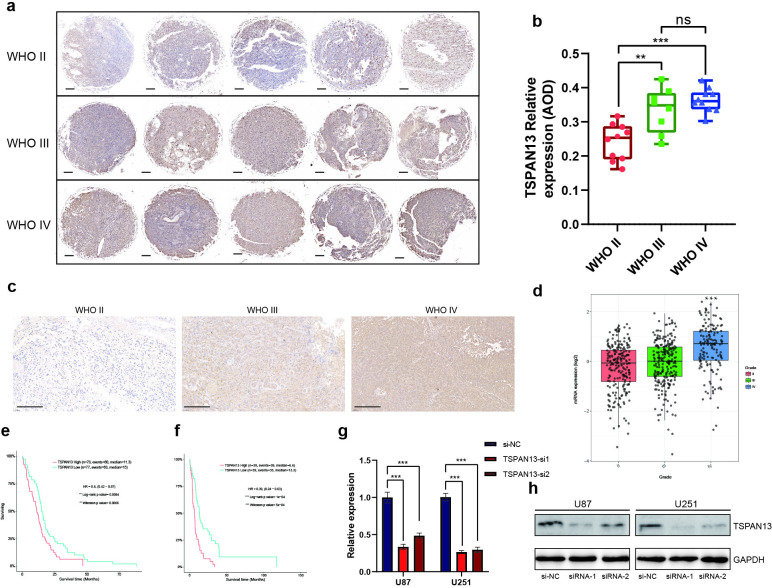
Upregulated TSPAN13 is a prognostic biomarker in glioma. (a) Representative pictures of IHC staining in our glioma tissue microarray cohort (scale bars of f =  200 μm). (b) TSPAN13 protein expression in the glioma tissue microarray. (c). Representative pictures of IHC staining in surgical removal samples (scale bars of g =  100 μm). (d) The expression levels of TSPAN13 in glioma tissues of different grades in the TCGA database. (e) K-M curves of TSPAN13 for GBM based on the TCGA-GBM cohorts. (f) K-M curves of TSPAN13 for GBM based on the Gravendeel cohorts. (g, h) TSPAN13 knockdown efficiency was detected using qRT-PCR and Western blotting in U251 and U87 cell lines. (*p <  0.05, **p <  0.01, ***p <  0.001, ns =  not significant).

On the basis of our previous study, we identified 12 candidate prognostic genes for GBM. Notably, most of these 12 prognostic TMZR-RDEGs, including IGFBP6 [34], BDKRB1 [35], SLC43A3 [36], MMP2 [37], HTRA1 [38], MDK [39], MME [40], PTX3 [41], PTPRN2 [42], and FLNC [43], have been reported to play critical roles in the development and invasiveness of GBM. However, the specific role of TSPAN13 in GBM has yet to be elucidated. However, multiple studies have shown the involvement of TSPAN13 in various human cancers, highlighting its important role [16-18,44]. Consequently, TSPAN13 was chosen for further experimental investigation. The expression level of TSPAN13 in glioma samples was further validated using immunohistochemical staining of a tissue microarray and fresh surgical removal of the tumor samples, with representative images displayed in Fig 6a, 6b and 6c. The results revealed that TSPAN13 protein expression was significantly lower in WHO grade II gliomas than in higher-grade gliomas (WHO III and IV) (Fig 6b). At the same time, analysis via the GlioVis database revealed that an increase in TSPAN13 expression correlated with an increase in glioma grade in the TCGA cohorts (Fig 6d) [45]. To explore the association between the TSPAN13 expression level and GBM prognosis, Kaplan‒Meier (KM) survival curves were generated using data from TCGA and Gravendeel GBM datasets. These analyses indicated a notable correlation between a high TSPAN13 expression level and a reduced overall survival time (OS) in patients, suggesting poorer outcomes, in both the TCGA and Gravendeel GBM cohorts (Fig 6e and 6f). To elucidate the biological function of TSPAN13 in glioblastoma multiforme (GBM) cells, we knocked down TSPAN13 in the U87 and U251 cell lines using two specific siRNAs. After transfection (72 hours), the TSPAN13 knockdown efficiency was verified through RT‒qPCR and western blotting (Fig 6g and 6h).

### Correlation of TSPAN13 expression with GBM cell proliferation, migration and tumor-related signaling pathways

**Fig 7:**
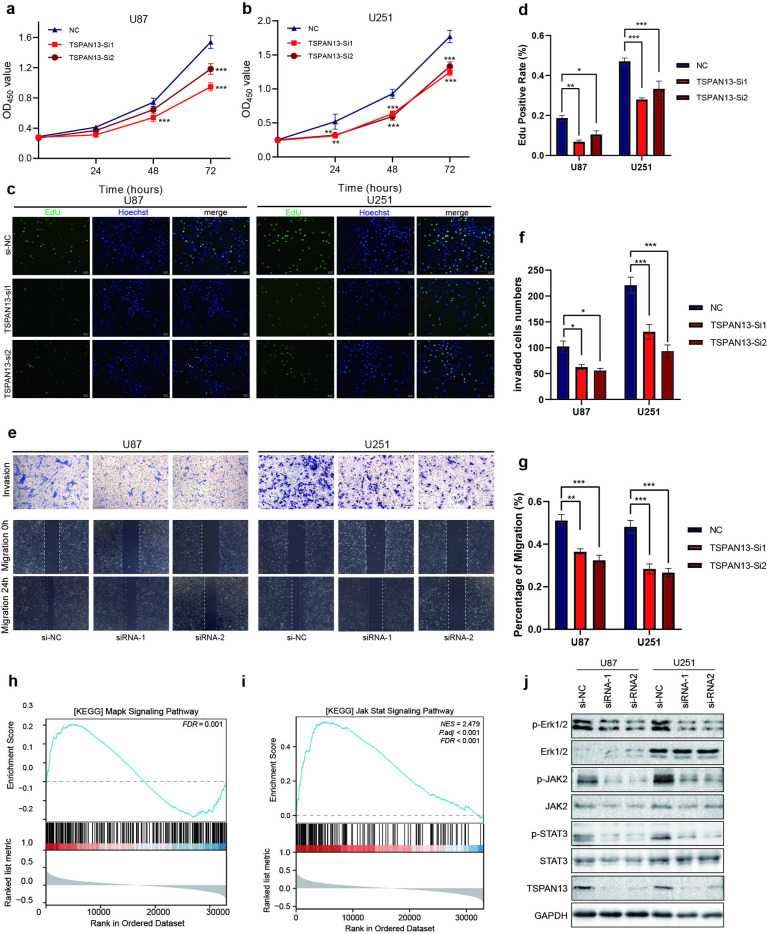
Knockdown TSPAN13 inhibits the proliferation, migration, and invasion abilities of GBM cells and suppresses the activation of the MAPK and JAK2/STAT3 signaling pathways. (a, b) Cell proliferation of U87 and U251 transfected with TSPAN13 siRNA and control was examined by CCK8 assay. (c) Representative pictures of EdU assays in U251 and U87 cell lines. (bar = 50 μm) (d) Statistical results of EdU assays in U251 and U87 cell lines. (e) Representative pictures of transwell and wound healing assays in U251 and U87 cell lines. (f, g) Statistical results of transwell and wound healing assays in U251 and U87 cell lines. (h, i) The GSEA of MAPK and JAK-STAT signalling pathway based on TSPAN13-associated genes. (j) Western blotting analysis showing the expression level of Erk1/2, p-Erk/2, JAK2, p-JAK2, STAT3 and p-STAT3 in TSPAN13 knockdown U87 and U251 cell lines. (*p < 0.05, **p < 0.01, ***p < 0.001).

We assessed the proliferation, invasion, and migration of glioma cells using conventional experimental methods. The results from the CCK-8 and EdU incorporation assays indicated that the knockdown of TSPAN13 markedly inhibited the proliferation of both U251 and U87 cells (Fig 7a, 7b, 7c, and 7d). Similarly, Transwell assays demonstrated that TSPAN13 knockdown significantly reduced the invasiveness of these cells (Fig 7e, 7f). Additionally, wound healing assays revealed that the migration ability of U251 and U87 cells was notably impeded following TSPAN13 knockdown (Fig 7e, 7g). We conducted Gene Set Enrichment Analysis (GSEA) of KEGG pathways for TSPAN13-associated genes. Some Pathways related to proliferation and temozolomide resistance, such as the MAPK and JAK-STAT signaling pathways (Fig 7h and 7i), were significantly enriched. We further verified the decreased activation of the ERK1/2 and JAK2-STAT3 signaling axes upon TSPAN13 knockdown using western blotting (Fig 7j). To examine the effects of TSPAN13 on the cell cycle, we conducted further analysis. Results revealed that TSPAN13 knockdown in U87 and U251 cells caused a substantial rise in the G0-G1 phase cell population, alongside a notable reduction in the S-phase population (S5a-d Fig). These findings suggest that TSPAN13 knockdown imposes an inhibitory effect on the glioma cell cycle. Taken together, TSPAN13 knockdown inhibited tumor proliferation and migration, as well as the activation of tumor-related signaling pathways.

### Downregulation of TSPAN13 enhanced the sensitivity of GBM to TMZ

**Fig 8:**
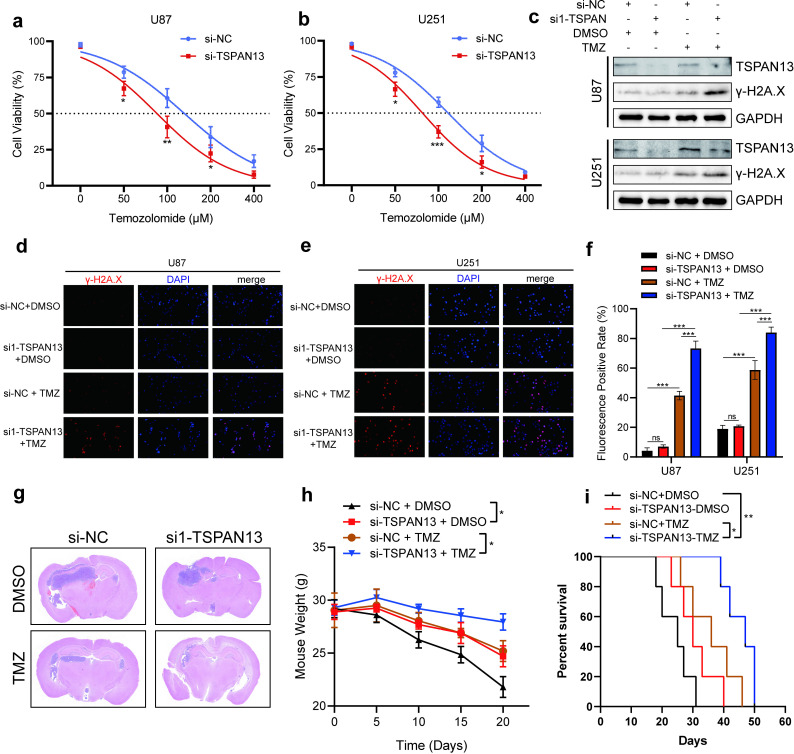
TSPAN13 knockdown can improve the sensitivity of GBM to temozolomide. (a, b) Cell viability of U87 and U251 treated with TMZ transfected with TSPAN13 siRNA and control was examined by CCK8 assay. (c-f) The expression of γ-H2A.X in U87 and U251 cells transfected with siRNA or treated with TMZ was detected by western blotting and immunofluorescence assay. (g) Representative HE-stained brain sections from tumor-bearing mice in each group of the animal model. (h). Weights of mice are recorded in each group of the animal model. (i). Kaplan–Meier survival of mice in each group. (*p <  0.05, **p <  0.01, ***p <  0.001, ns =  not significant).

We further examined the influence of TSPAN13 knockdown on the sensitivity of GBM cells to temozolomide. The CCK-8 assay results revealed that knocking down TSPAN13 significantly improved the efficacy of TMZ treatment in both the U87 and U251 cell lines (Fig 8a and 8b). γ-H2A.X, a marker indicative of DNA double-strand breaks in cells, is also an indicator of the cytotoxic effects of chemotherapeutic drugs [46]. Western blot analysis demonstrated a notable increase in the abundance of γ-H2A.X in TSPAN13-knockdown U87 and U251 cells following exposure to 100 μM temozolomide for 8 hours compared to that in the control siRNA-transfected cells (Fig 8c). These findings suggest that TSPAN13 knockdown substantially augments DNA damage in GBM cells treated with TMZ. The immunofluorescence assay results also showed increased expression intensity of γ-H2A.X in the nucleus after knocking down TSPAN13 (Fig 8d, 8e and 8f). To assess the potential role of TSPAN13 in influencing the efficacy of various antitumor drugs against glioma, we performed preliminary tests with drugs known for their cytotoxic effects on glioma. The drugs selected were carmustine [47], an alkylating agent; cisplatin [48], an apoptosis inducer; and gefitinib [49], an inhibitor targeting EGFR. The findings indicated that TSPAN13 knockdown increased glioma cell sensitivity to carmustine (S5e-f Fig), with no significant effects observed for cisplatin or gefitinib (S5g-j Fig). This heightened sensitivity to carmustine might stem from its similarity to TMZ, as both function as DNA alkylating agents with antitumor effects. These results offer valuable insights into the molecular role of TSPAN13 and its potential as a therapeutic target to overcome drug resistance in glioma.

To further validate the impact of TSPAN13 on tumor drug resistance in vivo, we constructed an orthotopic glioblastoma xenograft model. We found that knocking down TSPAN13 in tumor cells significantly increased their sensitivity to TMZ (Fig 8g). Compared to the control group and the TMZ monotherapy group, the weight loss was notably less severe (Fig 8h), and the overall survival time was significantly longer in the group with TSPAN13 knockdown combined with TMZ treatment (Fig 8i), further supporting the role of TSPAN13 in modulating TMZ sensitivity in GBM cells.

## Discussion

Temozolomide, a DNA-alkylating agent, is a first-line therapeutic option for glioblastoma. However, the resistance of glioma cells to temozolomide poses a significant challenge and limits the effectiveness of this treatment. Thus, investigating the mechanisms underlying temozolomide resistance holds substantial clinical importance [50]. In our study, we searched for DEGs across three distinct sets of data from temozolomide-resistant cell lines in the GEO database. To integrate the DEGs identified in these datasets, we employed the robust rank aggregation (RRA) algorithm. This approach enabled us to systematically integrate the findings from different data sources, providing a comprehensive analysis of the gene expression alterations associated with temozolomide resistance. To pinpoint crucial TMZR-RDEGs in GBM, we applied univariate Cox regression analysis and Cox regression analysis with the LASSO penalty. This process led to the identification of 12 TMZR-RDEGs, which were subsequently utilized to develop a TMZ resistance-related gene prognostic index (TMZR-RGPI) model for patients with GBM. The least absolute shrinkage and selection operator (LASSO) method, known for its penalization approach, was instrumental in shrinking and selecting variables for incorporation in the regression model [51]. The TMZR-RGPI model showed exceptional efficacy in predicting the prognosis of GBM patients. Indeed, the TMZR-RGPI score was found to be an independent prognostic factor intricately associated with both the clinicopathological and molecular characteristics of GBM. Furthermore, through stratified analysis, the model's effectiveness in differentiating prognoses for patients receiving temozolomide chemotherapy or radiotherapy was validated, with a higher TMZR-RGPI score consistently indicating a poorer prognosis. Furthermore, we developed a nomogram incorporating the TMZR-RGPI score, improving the applicability of the model in clinical settings. This advancement highlights our model's potential as a valuable predictive tool that is especially useful in formulating treatment strategies and conducting prognostic assessments for GBM patients. This finding not only reaffirms the model's clinical relevance but also points towards its utility in guiding personalized treatment approaches in GBM management.

In our investigation of the association between the TMZR-RGPI and genetic mutations in GBM, we observed distinct patterns. TP53 mutations were found to be the most common mutations in both the high- and low-TMZR-RGPI subgroups. Notably, mutations in TTN, SPTA1, RYR2, and PKHD1 were more common in the high-TMZR-RGPI subgroup. In contrast, TP53, RB1, FLG, and ATRX mutations were more common in the low-TMZR-RGPI subgroup. Additionally, the tumour mutational burden (TMB), which can indirectly indicate neoantigen production and predict immunotherapy efficacy in various cancers, was explored. A higher TMB has been associated with improved overall survival (OS) and a more favourable response to immune checkpoint inhibitor (ICI) therapy across most cancer types. Our findings revealed a negative correlation between the TMZR-RGPI score and the TMB, providing indirect evidence of the prognostic utility of the TMZR-RGPI. These insights may contribute to a deeper understanding of GBM pathogenesis and have potential implications for patient treatment strategies.

Our functional enrichment and immune cell infiltration analyses revealed that our risk model was significantly correlated with various biological processes and pathways in GBM. These included response to chemical, inflammatory response, extracellular matrix organization, pro-cancer-related pathways, immune-related pathways, and infiltration of immune cells in GBM tumours. Consequently, the 12 genes identified through our model are potential therapeutic targets for GBM. The significant associations of these genes with these key biological processes and pathways underscore their potential role in GBM pathophysiology and suggest new avenues for therapeutic intervention.

Indeed, some of these 12 genes have been reported to be involved in gliomagenesis or to be significant predictors of overall survival (OS). For instance, C. R. Oliva et al. reported that IGFBP6 regulates temozolomide (TMZ) resistance in glioblastoma through its paracrine effects on IGF2/IGF-1R signalling [34]. MMP2 is known to influence glioma angiogenesis and enhance tumour proliferation and invasion [52]; thus, it is a key biomarker for the response to antivascular therapy [53]. IGFBP2 has been shown to increase glioma angiogenesis by upregulating MMP2 expression [54]. BDKRB1, or Bradykinin Receptor B1, has been implicated in promoting the activation of tumour-associated macrophages, thereby facilitating tumour progression and migration [55]. According to Yu et al., high MDK expression in GBM contributes to increased cell proliferation and stemness and promotes TMZ resistance through the activation of the Notch1/p-JNK signalling pathway [56]. PTX3, an important immunomodulatory molecule in glioma, has been reported to enhance macrophage infiltration and migration and to promote the malignant transformation of dendritic cells in the tumour microenvironment [41,57]. Furthermore, high FLNC expression in glioma is associated with poor patient prognosis, increased invasion capacity, and elevated MMP2 expression [43]. These studies collectively reinforce the importance of the identified risk model. Given the important roles these genes play in GBM, the remaining TMZR-RDEGs that have yet to be studied extensively in the context of glioma constitute valuable objects for future research.

Among the 12 TMZR-RDEGs identified in our study, TSPAN13 was found to be the only gene not previously reported in glioma research. Therefore, we focused our subsequent investigations on validating the role of TSPAN13 in glioma progression and drug resistance. Recent studies have increasingly highlighted the importance of TSPAN13 in various human cancers, as it is strongly correlated with poor patient prognosis. TSPAN13 belongs to the transmembrane 4 superfamily, which is involved in mediating signal transduction processes that are essential for the regulation of cell development, activation, growth, and motility [44]. In prostate cancer, studies have found that TSPAN13 is highly expressed in 80% of prostate cancer samples, suggesting that it may play a promotive role in the development of prostate cancer [58]. Research has found that downregulation of TSPAN13 by miR-369-3p can inhibit the proliferation of papillary thyroid cancer cells [59]. Similarly, studies have shown that miR-4732-5p promotes breast cancer progression by targeting TSPAN13 [18]. In spatial transcriptomics studies of colorectal cancer, molecules including TSPAN13 were found to be specifically highly expressed in tumor regions, indicating their potential roles in tumor development [60]. Despite these insights, the specific role of TSPAN13, one of the 12 TMZR-RDEGs, in GBM has yet to be elucidated. To address this knowledge gap, we focused our investigation on the role of TSPAN13 in GBM. Through bioinformatic analysis, we discovered that TSPAN13 is aberrantly upregulated in GBM tissues and is significantly associated with poor prognosis in primary GBM patients. We observed that an increase in TSPAN13 expression correlated with an increase in the grade of glioma. These findings were further corroborated by our tissue microarray immunohistochemistry results, confirming the potential identity of TSPAN13 as a key player in GBM pathogenesis and a promising target for future research.

In our study, we confirmed that knockdown of TSPAN13 in GBM cell lines significantly reduced cell proliferation, cell cycle, migration, and invasion in vitro. Through GSEA enrichment analysis, we found that TSPAN13 is associated with multiple tumor-related signaling pathways. Experimental validation demonstrated that knocking down TSPAN13 can inhibit the activation of the MAPK and JAK2/STAT3 pathways. These findings suggest that TSPAN13 plays a crucial role in the aggressive behaviour of GBM cells. Furthermore, we observed that TSPAN13 knockdown enhanced the sensitivity of GBM cells to temozolomide, a key chemotherapeutic agent used in GBM treatment. We investigated the DNA double-strand break marker γ-H2A.X through western blotting and immunofluorescence staining. Further experiments revealed that TSPAN13 knockdown increased glioma cell sensitivity to carmustine, another alkylating agent, while showing no significant impact on sensitivity to the apoptosis inducer cisplatin or the EGFR inhibitor gefitinib. In vivo orthotopic mouse xenograft models also confirmed that knocking down TSPAN13 significantly increases the tumor's sensitivity to temozolomide. Our results revealed that TSPAN13 knockdown indeed increased TMZ-induced DNA damage in glioma cells and inhibited the TMZ resistance of GBM in vivo.

Despite the promising findings of our study, it is important to acknowledge certain limitations. While our study primarily focused on identifying TSPAN13 as a novel prognostic marker for TMZ resistance in glioma, further research is needed to determine the underlying mechanisms and regulatory pathways associated with TSPAN13 in more detail. A deeper exploration of these aspects would provide a more comprehensive understanding of the role of TSPAN13 in GBM and its potential as a therapeutic target.

## Conclusion

In this study, we successfully developed a prognostic model incorporating 12 TMZR-RDEGs, which demonstrated excellent efficacy in predicting the prognosis of GBM patients. This model not only offers prognostic insights but also reveals crucial molecular and immunological characteristics associated with GBM, highlighting potential therapeutic targets for GBM treatment. A key finding of our research is the important role of TSPAN13: its downregulation in GBM cells suppresses tumour proliferation, migration, invasion, and resistance to TMZ. These results position TSPAN13 as a promising therapeutic target in GBM, particularly for modulating sensitivity to TMZ. Our study thus provides valuable insights into GBM treatment strategies and opens avenues for future therapeutic development.

## Supporting information

S1 Fig Validation of TMZR-RGPI model for GBM. (a, d) Kaplan–Meier curves for OS in the CGGA693-GBM and CGGA325-GBM cohort stratified by 12 TMZR-RDEGs model in high- and low-risk. (b, e) The distribution plots of TMZR-RGPI, survival status and expression of 12 selected TMZR-RDEGs in the CGGA693-GBM and CGGA325-GBM cohort. (c, f) Time dependent ROC curves for TMZR-RGPI model in the CGGA693-GBM and CGGA325-GBM cohort.
(TIF)

S2 Fig Stratified OS analysis with different clinicopathological characteristics in the TCGA-GBM, CGGA693-GBM and CGGA325-GBM cohort. (a, b) Stratification analysis according to MGMT promoter status. Kaplan‒Meier curves showed survival differences between the high and low TMZR-RGPI subgroups. (c, d) The OS between high and low TMZR-RGPI subgroups in patients with/without chemotherapy. (e, f) The OS between high and low TMZR-RGPI subgroups in patients with/without radiotherapy.
(TIF)

S3 Fig The mutation profile and TMB of different TMZR-RGPI subgroups. (a) Mutation profile in high and low TMZR-RGPI subgroups. (b) Association between TMB and TMZR-RGPI and its distribution in the low and high TMZR-RGPI subgroups.
(TIF)

S4 Fig Functional Enrichment Analysis of TMZR-RGPI. (a) Volcano plot displaying differentially expressed protein-coding genes between high- and low-TMZR-RGPI groups in the TCGA-GBM dataset. (b) Top 10 biological process terms from GO enrichment analysis of 205 DEGs. (c) Top 10 cellular component terms from GO enrichment analysis of 205 DEGs. (d) Top 10 molecular function terms from GO enrichment analysis of 205 DEGs. (e) GSEA enrichment plot for KEGG gene sets. f GSEA enrichment plot for HALLMARK gene sets.
(TIF)

S5 Fig Effects of TSPAN13 knockdown on glioma cell cycle progression and resistance to other anticancer agents. (a-d) Flow cytometry analysis showing changes in cell cycle distribution following TSPAN13 knockdown in U87 cells (a) and U251 cells (c), with corresponding statistical results (b, d). (e-j) CCK8 assay evaluating the impact of TSPAN13 knockdown on resistance to other anticancer drugs in U87 and U251 cells, including carmustine (e, f), cisplatin (g, h), and gefitinib (i, j).
(TIF)

S1 Table DEGs identified using RRA methods. (XLSX)

S2 Table The 48 prognositc TMZR-RDEGs based on the TCGA GBM cohort. (XLSX)

S3 Table The results of GO enrichment analysis. (XLSX)

S1_raw_images All original immunoblot images. (PDF)
