# Supplementary figures and images for: Identification and validation of TSPAN13 as a novel temozolomide resistance-related gene prognostic biomarker in glioblastoma
Source: PLoS One. 2025 Feb 4;20(2):e0316552. doi: 10.1371/journal.pone.0316552 (PMC11793784; doi:10.1371/journal.pone.0316552)

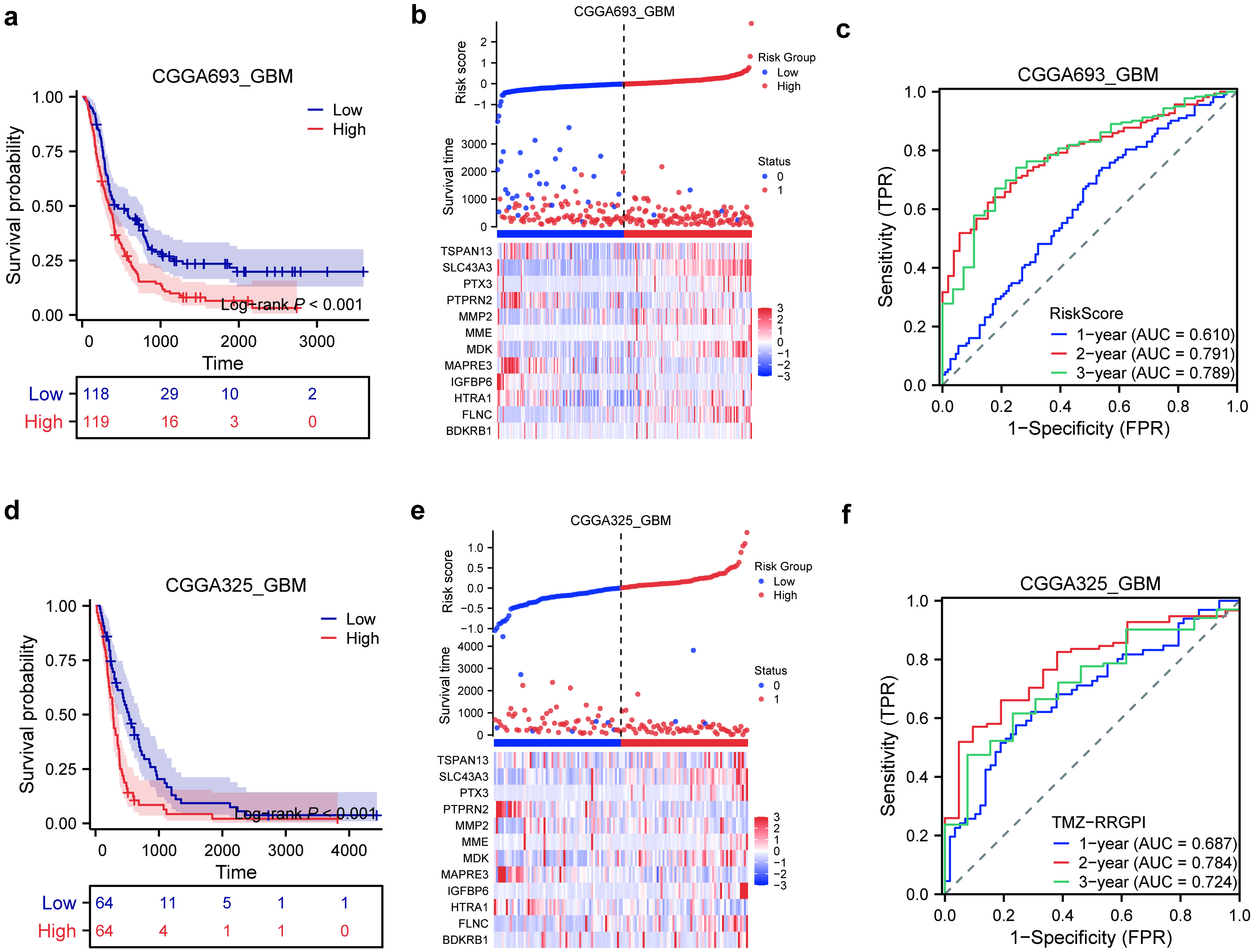

Supplement: S1 Fig — (a, d) Kaplan–Meier curves for OS in the CGGA693-GBM and CGGA325-GBM cohort stratified by 12 TMZR-RDEGs model in high- and low-risk. (b, e) The distribution plots of TMZR-RGPI, survival status and expression of 12 selected TMZR-RDEGs in the CGGA693-GBM and CGGA325-GBM cohort. (c, f) Time dependent ROC curves for TMZR-RGPI model in the CGGA693-GBM and CGGA325-GBM cohort. (TIF) [file pone.0316552.s001.tif]

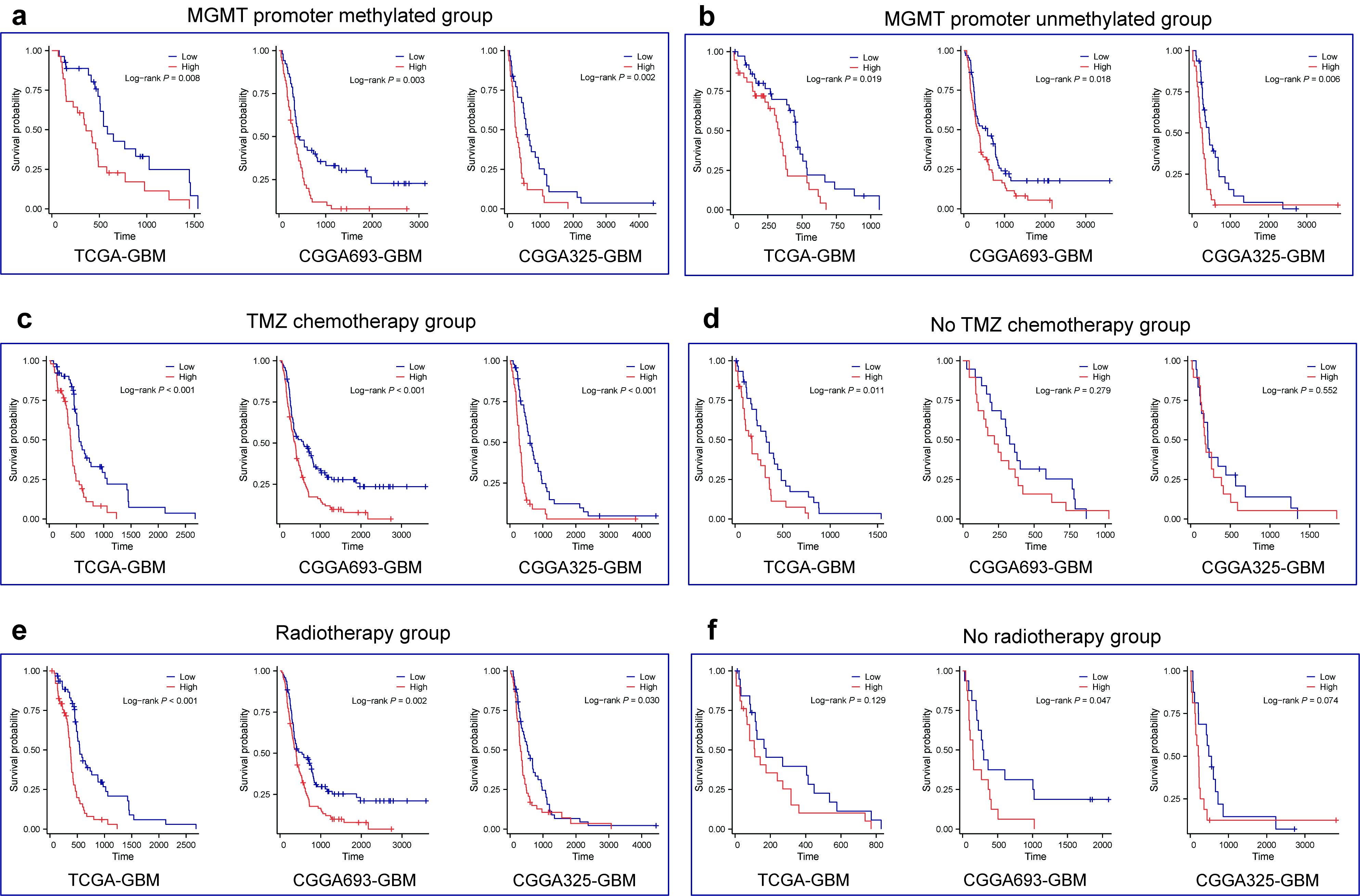

Supplement: S2 Fig — (a, b) Stratification analysis according to MGMT promoter status. Kaplan‒Meier curves showed survival differences between the high and low TMZR-RGPI subgroups. (c, d) The OS between high and low TMZR-RGPI subgroups in patients with/without chemotherapy. (e, f) The OS between high and low TMZR-RGPI subgroups in patients with/without radiotherapy. (TIF) [file pone.0316552.s002.tif]

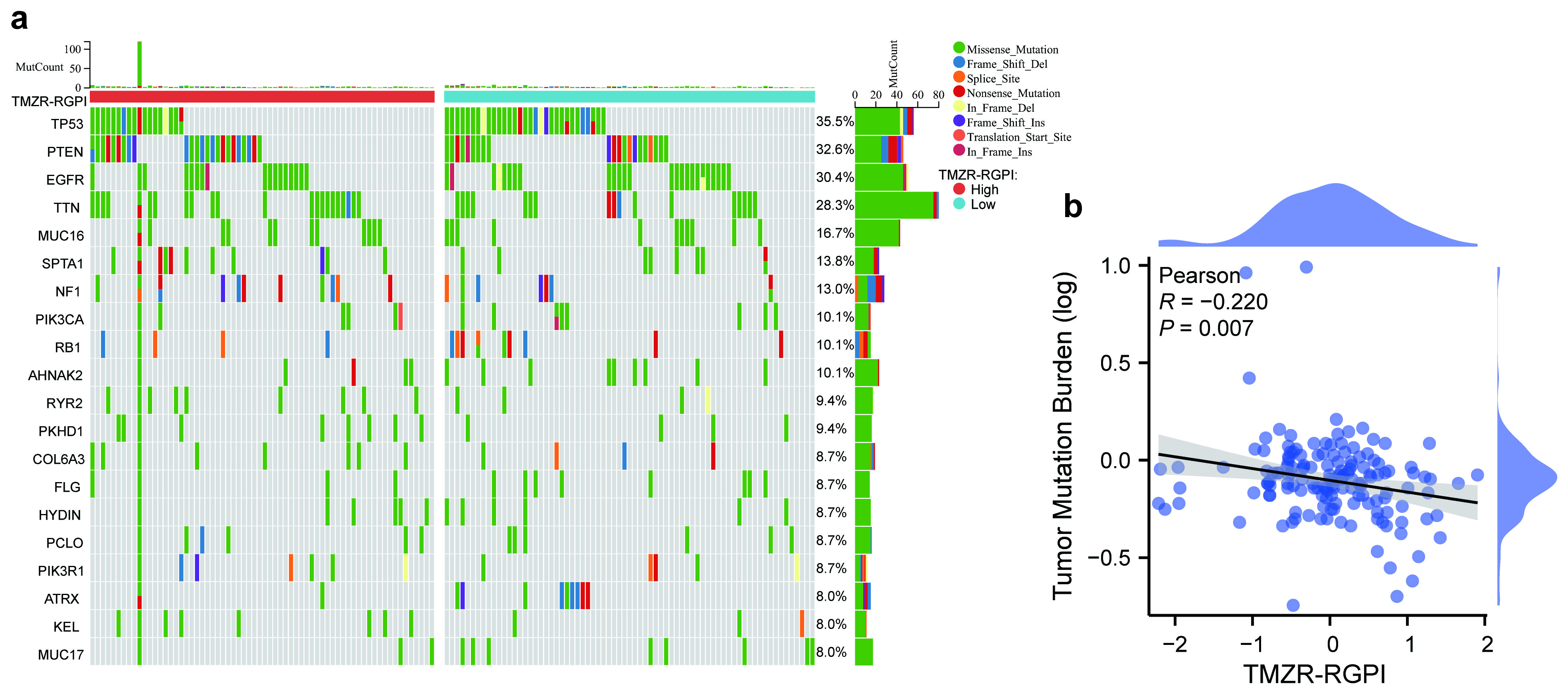

Supplement: S3 Fig — (a) Mutation profile in high and low TMZR-RGPI subgroups. (b) Association between TMB and TMZR-RGPI and its distribution in the low and high TMZR-RGPI subgroups. (TIF) [file pone.0316552.s003.tif]

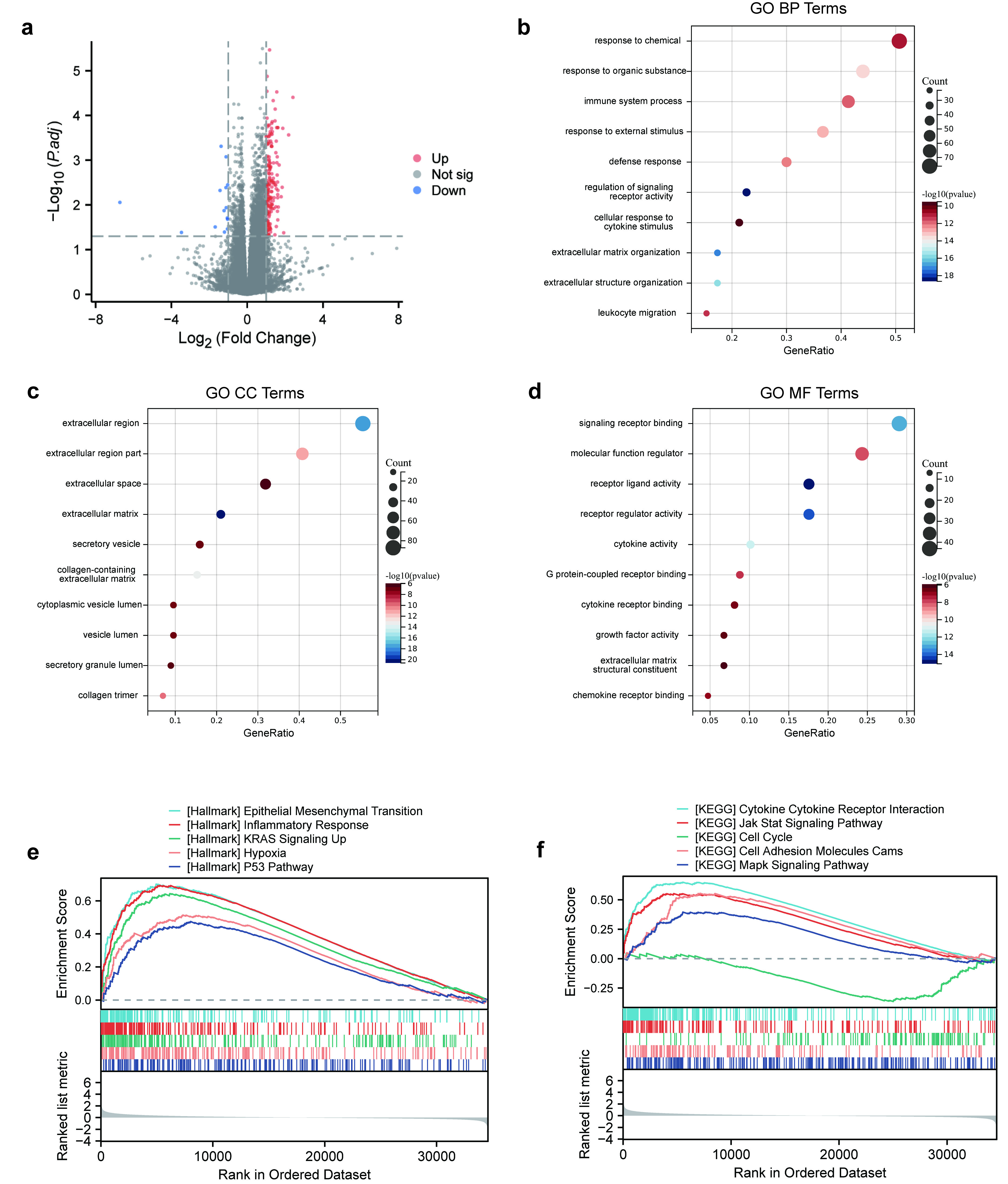

Supplement: S4 Fig — (a) Volcano plot displaying differentially expressed protein-coding genes between high- and low-TMZR-RGPI groups in the TCGA-GBM dataset. (b) Top 10 biological process terms from GO enrichment analysis of 205 DEGs. (c) Top 10 cellular component terms from GO enrichment analysis of 205 DEGs. (d) Top 10 molecular function terms from GO enrichment analysis of 205 DEGs. (e) GSEA enrichment plot for KEGG gene sets. f GSEA enrichment plot for HALLMARK gene sets. (TIF) [file pone.0316552.s004.tif]

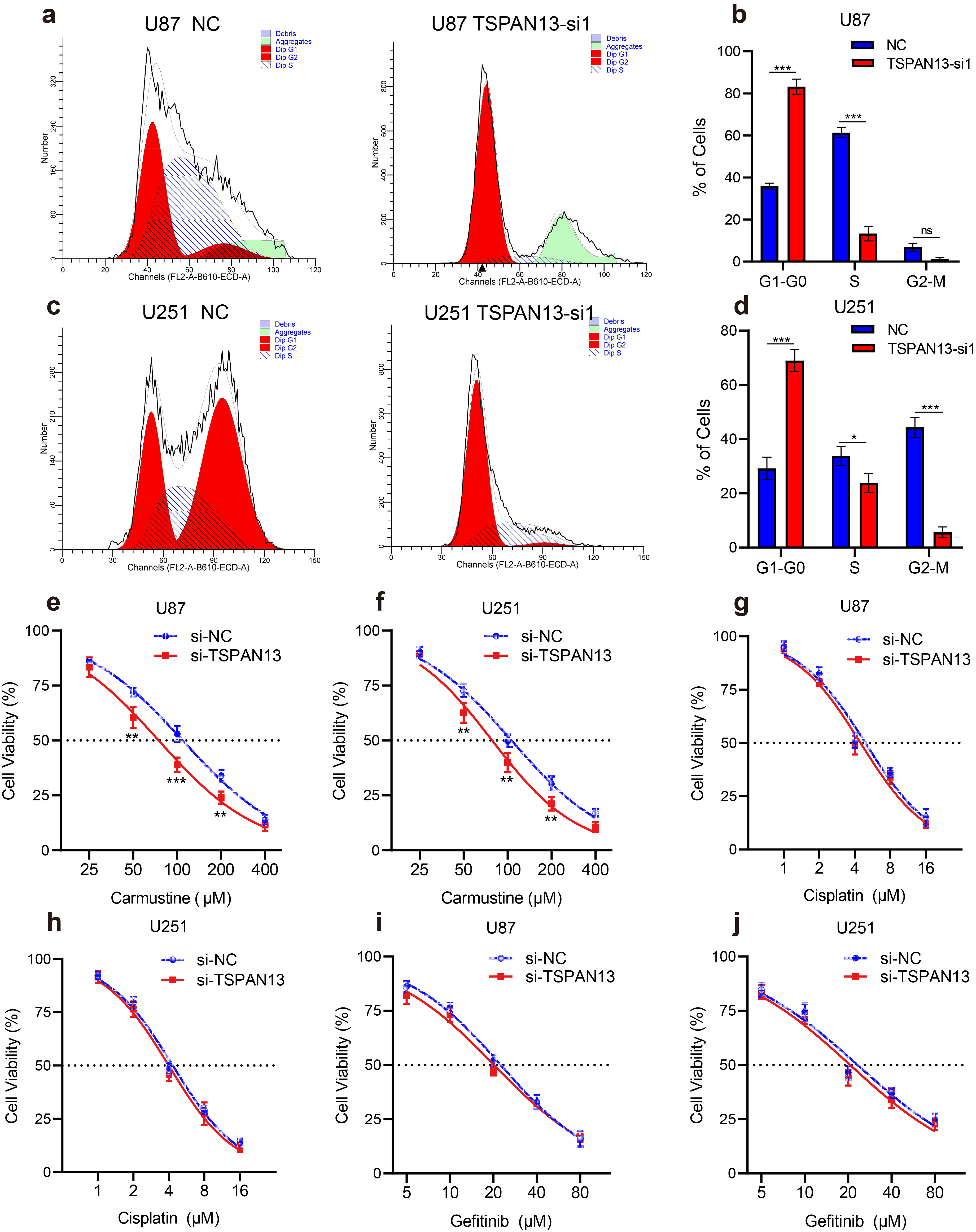

Supplement: S5 Fig — (a-d) Flow cytometry analysis showing changes in cell cycle distribution following TSPAN13 knockdown in U87 cells (a) and U251 cells (c), with corresponding statistical results (b, d). (e-j) CCK8 assay evaluating the impact of TSPAN13 knockdown on resistance to other anticancer drugs in U87 and U251 cells, including carmustine (e, f), cisplatin (g, h), and gefitinib (i, j). (TIF) [file pone.0316552.s005.tif]

Figure 6h

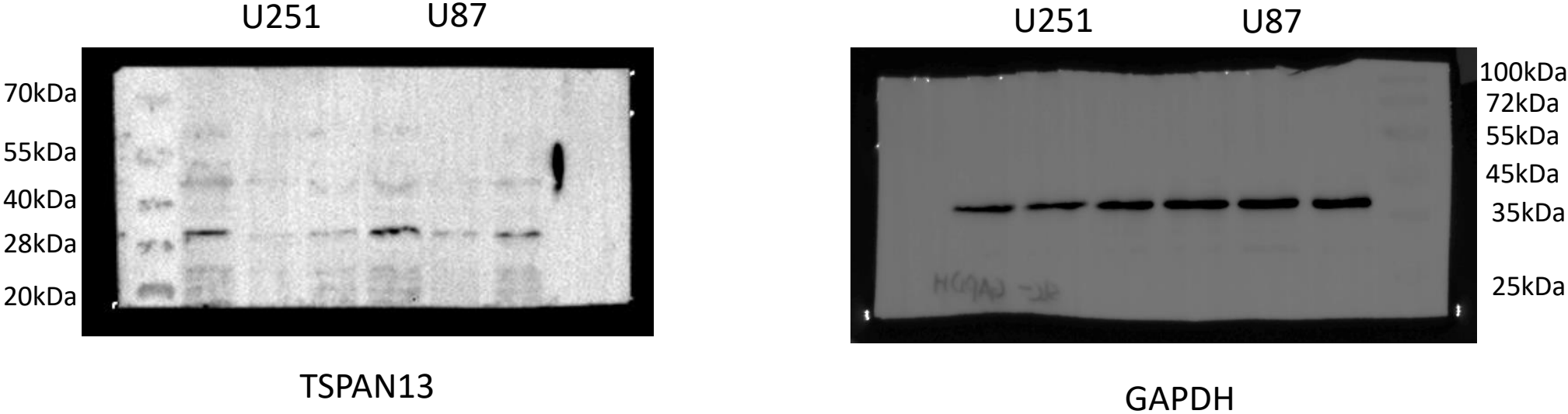

Figure 7j

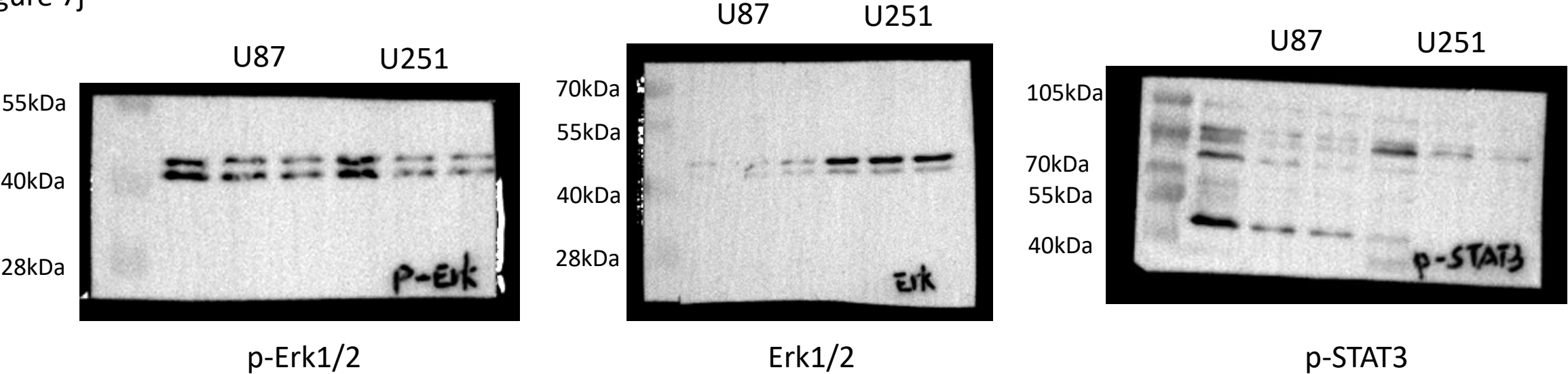

Figure 7j

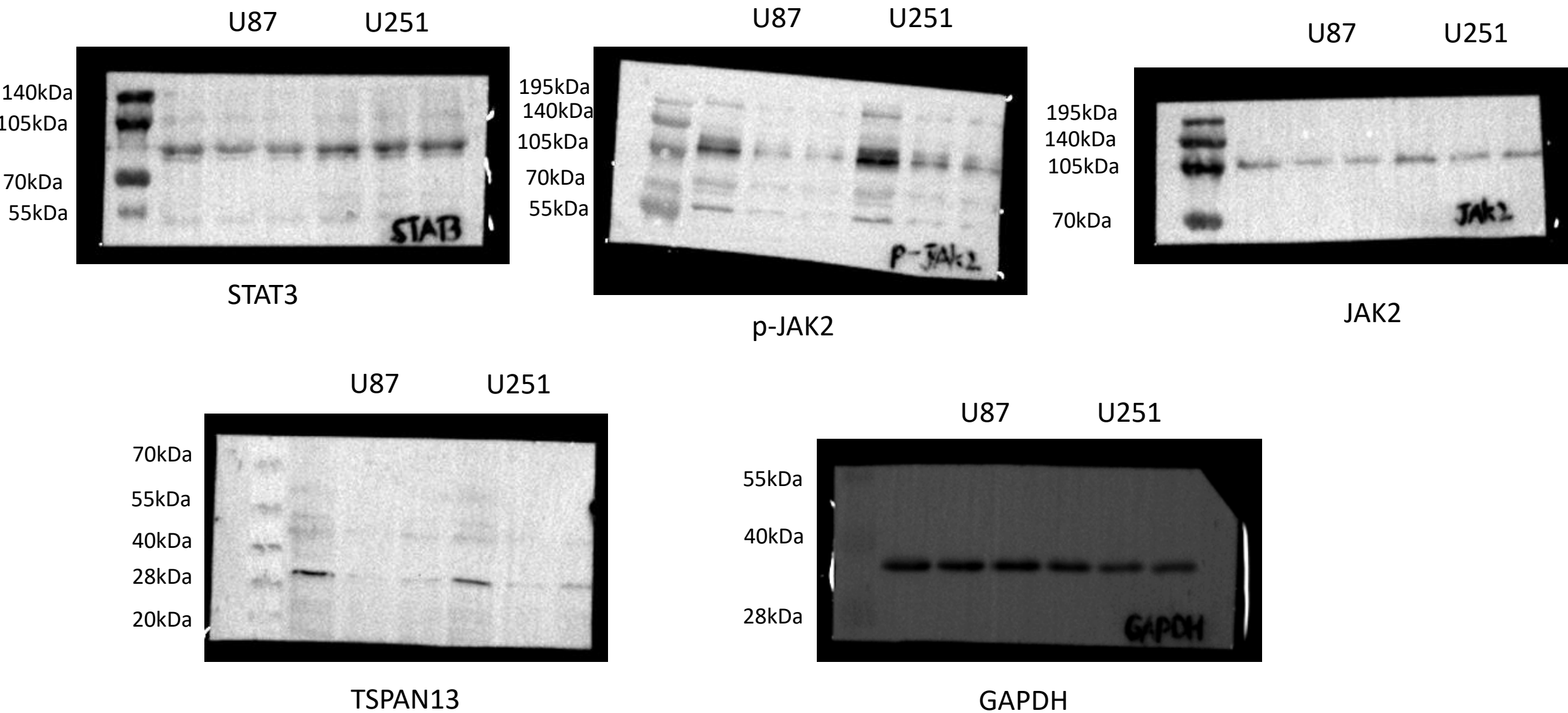

Figure 8c

U87

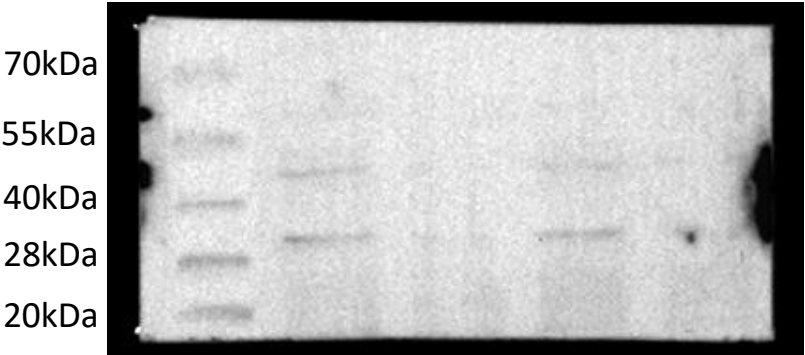

TSPAN13

U251

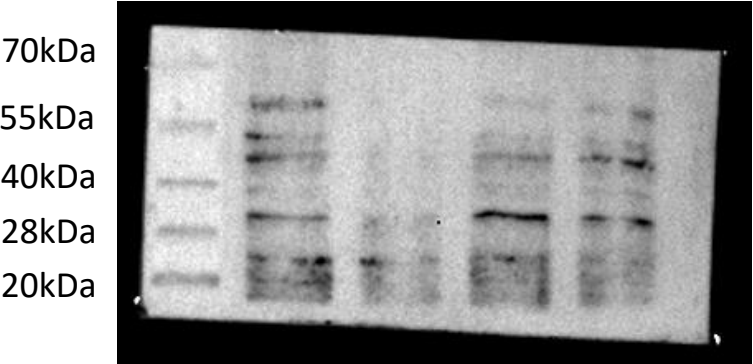

TSPAN13

U251

U87

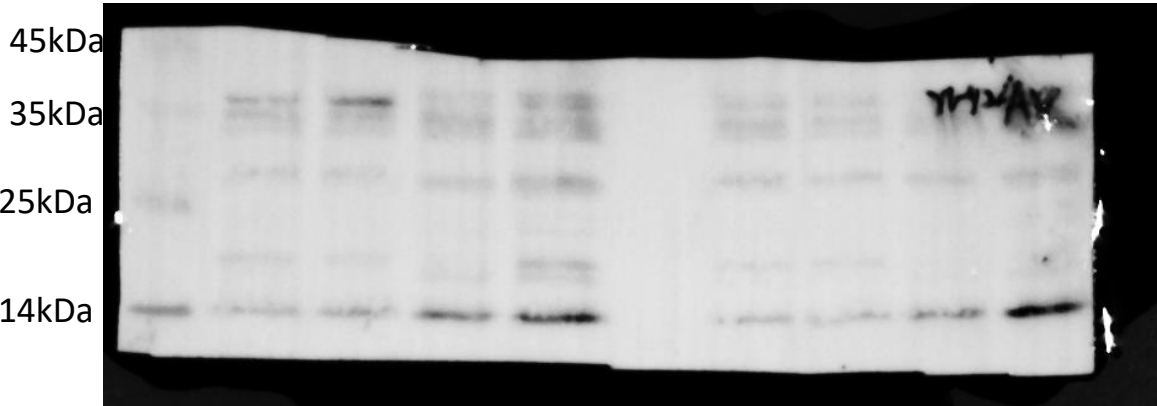

γ-H2AX

U251

U87

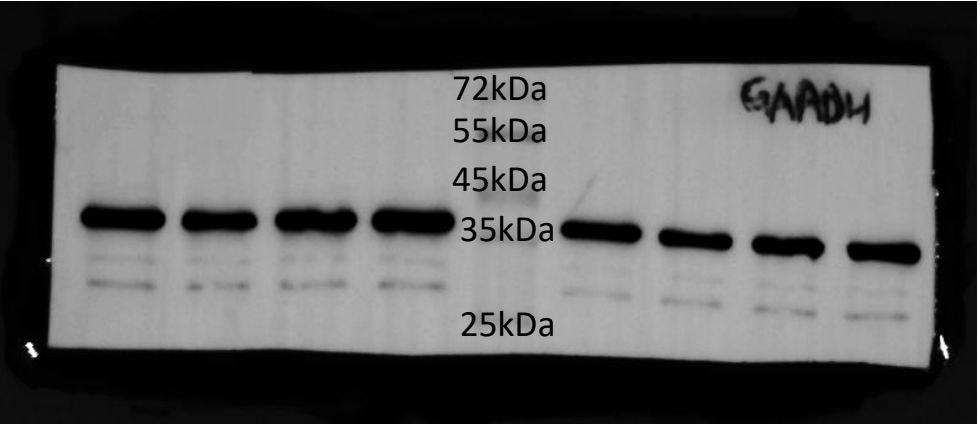

GAPDH

Supplement: S1_raw_images — (PDF) [file pone.0316552.s009.pdf]
